# An efficient interpretable framework for unsupervised low, very low and extreme birth weight detection

**DOI:** 10.1371/journal.pone.0317843

**Published:** 2025-01-30

**Authors:** Ali Nawaz, Amir Ahmad, Shehroz S. Khan, Mohammad Mehedy Masud, Nadirah Ghenimi, Luai A. Ahmed

**Affiliations:** 1 College of Information Technology, UAEU, Al Ain, UAE; 2 College of Engineering and Technology, American University of the Middle East, Dasman, Kuwait; 3 College of Medicine and Health Sciences, UAEU, Al Ain, UAE; Khalifa University, UNITED ARAB EMIRATES

## Abstract

Detecting low birth weight is crucial for early identification of at-risk pregnancies which are associated with significant neonatal and maternal morbidity and mortality risks. This study presents an efficient and interpretable framework for unsupervised detection of low, very low, and extreme birth weights. While traditional approaches to managing class imbalance require labeled data, our study explores the use of unsupervised learning to detect anomalies indicative of low birth weight scenarios. This method is particularly valuable in contexts where labeled data are scarce or labels for the anomaly class are not available, allowing for preliminary insights and detection that can inform further data labeling and more focused supervised learning efforts. We employed fourteen different anomaly detection algorithms and evaluated their performance using Area Under the Receiver Operating Characteristics (AUCROC) and Area Under the Precision-Recall Curve (AUCPR) metrics. Our experiments demonstrated that One Class Support Vector Machine (OCSVM) and Empirical-Cumulative-distribution-based Outlier Detection (ECOD) effectively identified anomalies across different birth weight categories. The OCSVM attained an AUCROC of 0.72 and an AUCPR of 0.0253 for extreme LBW detection, while the ECOD model showed competitive performance with an AUCPR of 0.045 for very low LBW cases. Additionally, a novel feature perturbation technique was introduced to enhance the interpretability of the anomaly detection models by providing insights into the relative importance of various prenatal features. The proposed interpretation methodology is validated by the clinician experts and reveals promise for early intervention strategies and improved neonatal care.

## 1 Introduction

According to the World Health Organization (WHO), over 20 million babies are born with low birth weight (LBW) each year. [1]. A newborn is considered to have LBW if the weight is less than 2,500 grams. Very LBW applies to newborns weighing less than 1,500 grams, and extreme LBW refers to newborns weighing under 1,000 grams [2]. LBW presents significant neonatal and public health challenges due to its high morbidity and mortality rates [3]. Infants with LBW are more vulnerable to complications such as infections, respiratory problems, and feeding difficulties. LBW also raises the risk of developing chronic illnesses such as cardiovascular disease and diabetes later in life [4, 5]. Therefore, addressing LBW is critical to improving infant survival, quality of life, and lowering healthcare expenditures. Effective and early detection and prediction are critical for mitigating long-term effects of LBW [6].

Artificial intelligence (AI) is becoming increasingly valuable in neonatal care, particularly in predicting birth weights, a critical factor in newborn health. AI utilizes historical data by considering various neonatal and maternal health indicators to detect potential low or high birth weight outcomes [7, 8]. AI helps in finding hidden patterns and correlations that are difficult for clinicians to understand directly [9]. These insights can more accurately estimate birth weight by allowing more proactive management and personalized care strategies during pregnancy.

Anomaly detection is the process of identifying unexpected events in the dataset which are different from normal [10]. The diagnosis of medical condition during pregnancies are critical because it can trigger additional medical evaluation and care for high-risk pregnancies [11]. Furthermore, unsupervised learning models in anomaly detection may excel at dealing with the unpredictability and rarity of extreme birth weight events, which are frequently underrepresented in datasets [12]. By using these models, clinicians can detect tiny irregularities in fetal development and offering a more comprehensive insight of each pregnancy and increasing the possibility of early and effective intervention.

Specifically, we have applied a variety of unsupervised anomaly detection approaches on private collected datasets to detect LBW, VLBW, and ELBW separately. Through the applied approach, we observed that the models perform better when identifying extreme cases and imbalance nature of data. Furthermore, we have presented a novel technique that modifies specific data instances to improve the interpretability of anomaly scores. The proposed technique helps us to understand the contribution of each feature to the output of the model.

### 1.1 Motivation

The proposed methodology is effective for the limited data available early in pregnancy as finding pattern in small datasets is difficult than big data [13]. By recognizing essential patterns early, we can inform interventions that may include planning nutritional diets during pregnancy [14]. Additionally tabular data is simple to obtain can be used as an early indicator of expected risks of birth weight [15, 16]. The proposed methodology is critical for enhancing neonatal care via proactive and focused healthcare initiatives [17]. Therefore, it may be more effective to handle the problem as unsupervised anomaly detection rather than binary class classification problem [18].

The main contributions of research paper are summarised as;

- The collected datasets is ambiguous therefore through careful refinement, we addressed inconsistencies and prepared the dataset for robust analytical exploration by ensuring accuracy and reliability for further experimentation.
- We employed multiple unsupervised anomaly detection models on preprocessed datasets and our experimentation demonstrates improved detection performance as the proportion of minority points decreases from low towards extreme birth weight.
- A novel feature perturbation technique is introduced to provide interpretability specifically for anomaly detection scores. The proposed technique offers insights into the contribution of individual features on the anomaly score aiding in the understanding of complex models and contributing to evidence-based decision-making of anomaly detection models.
- The proposed approach is particularly well-suited to the often sparse datasets available in early pregnancy by providing a framework for identifying and understanding critical patterns that can inform early interventions.

The remainder of this paper is structured as follows: Section II presents a literature review of the applied anomaly detection models, interpretation techniques, and related work on LBW. The proposed methodology is presented in Section III. Experimental results is shown in Section IV. Finally, Section V concludes the paper.

## 2 Literature review

In this section, review of literature on anomaly detection models, interpretation techniques, and LBW detection will be explained in detail.

### 2.1 Anomaly detection models

Anomaly detection techniques have been used to identify neonatal factors associated with LBW and other adverse pregnancy outcomes:

The study by Massara et al. [19] used clustering-based outlier detection methods like the Multi-Model Outlier Measurement Detection (MMOM) approach to identify synthetic outliers in infant weight and length measurements. The MMOM method was able to better detect milder outliers compared to other approaches. Additionally, a node embedding-based graph autoencoder (GAE) outlier detection algorithm to predict adverse pregnancy outcomes such as low birth weight and preterm birth was proposed by Khan et al. [20]. By incorporating complex relational structures between patients in a knowledge graph, the GAE model demonstrated improved performance over traditional machine learning approaches while handling class imbalance problem in medical datasets.

Gao et al. [21] applied a “Triple O+” (Outlier-based Outcome Prediction) approach to detect neonatal brain connectivity outliers and demographic outliers (based on maternal education, birth weight, and gestational age) to predict 4-year-old IQ outliers. This approach was able to identify 42.1% of 4-year-old IQ outliers with 96.2% specificity and 90.3% overall accuracy, demonstrating the potential of brain-based outlier detection for early identification of children at risk of poor cognitive outcomes. Another experiment by Janoudi et al. [22] explored the use of augmented intelligence, including outlier analysis methods like extreme misclassification and isolation forest, to accelerate the discovery of novel clinical insights related to preeclampsia and hypertensive disorders of pregnancy. The extreme misclassification approach identified a higher proportion of potential novelties compared to the traditional isolation forest method. Additionally, an experimentation by Bardwell et al. [23] employed anomaly detection has been used to establish reference intervals for fetal and neonatal intestinal lengths, which is important for identifying abnormalities during postmortem examinations. Proper statistical methods, including partitioning by gestational age and sensitivity analysis, are crucial for accurately defining these reference ranges and detecting outliers.

Röchner et al. [24] investigates unsupervised methods to identify questionable electronic health records (EHRs) in cancer registries. It uses 21,104 records from a German cancer registry and employed techniques such as Frequent Pattern Outlier Factor (FindFPOF) and autoencoder. The findings suggest that both methods detected around 28% of EHRs as implausible, compared to 8% by random selection. The proposed methods exhibited same specificity of 94% but improved sensitivity of 22% and 26% for autoencoder and FPOP respectively.

Additionally, a recent advancements in anomaly detection have focused on developing robust models for biomedical and healthcare applications. Akbar et al. [25] created a Deepstacked-AVPs model that finds antiviral peptides using tri-segment matrices and word2vec. It predicts accurately and aids in antiviral drug screening due to its clarity and stability. Rukh et al. [26] developed StackedEnC-AOP, which uses multi-scale features from evolutionary and sequential data to identify antioxidant proteins. By layering multiple models, it achieves higher accuracy and benefits antioxidant research.

Anomaly detection techniques applied to neonatal brain connectivity, demographic factors, and other clinical measurements show promise for identifying at-risk infants and accelerating clinical discoveries related to adverse pregnancy outcomes.

### 2.2 Interpretation techniques

Interpretation techniques in ML offer insights into the decision-making process of models while enhancing their transparency and trust. SHAP (SHapley Additive exPlanations) [27] and LIME (Local Interpretable Model-agnostic Explanations) [28] are two prominent and widely used interpretation technique. SHAP assigns an importance value to each feature and reveal the feature impact on the output of the model. LIME on the other hand, approximates the model locally and provides interpretation for individual predictions. However, both of these methods are computationally expensive [29]. Therefore, we will compare our proposed interpretation technique with Depth-based feature importance of IF (Local-DIFFI) [30] due to its computational efficiency.

Local-DIFFI technique enhances interpretability in IF models by quantifying the impact of each feature on the decision-making process of the model [30]. By leveraging the structural insights provided by IFs, Local-DIFFI assesses how alterations in feature values influence the path lengths of data points within the trees, offering a subtle view of feature contributions towards the anomaly score [31]. The method not only aids in understanding which features drive anomalies but also facilitates a deeper analysis of underlying structure of data by promoting informed decision-making in complex scenarios. The Local-DIFFI method for IFs provides an interpretative framework to assess the impact of each feature on the anomaly score. It is defined as follows:

Given an isolation tree *T* and a datapoint *x*, the anomaly score is influenced by the path length *h*(*x*, *T*). Local-DIFFI quantifies the change in *h*(*x*, *T*) as a function of feature perturbation. For feature *i*, the importance *I*_*i*_ is computed by Eq (1):

$$\begin{array}{c} I_{i}\left(x\right)=|h\left(x,T\right)-h\left(x_{i}^{\prime },T\right)| \end{array}$$ (1)

where $x_{i}^{\prime }$ represents the data point *x* with the *i*^*th*^ feature replaced by a perturbed value (e.g., the mean of feature *i* across the training set). The depth difference highlights the feature’s influence on the isolation process, with larger values indicating higher importance.

By averaging *I*_*i*_(*x*) over all trees in the forest, we obtain a comprehensive measure of feature importance across the model, enabling insights into the features most indicative of anomalies.

AI and machine learning models have been used to predict various perinatal health indicators like preterm birth, low birthweight, preeclampsia, and postpartum depression [32]. However, for these AI models to be widely adopted in clinical practice, it is crucial to make them explainable so that healthcare providers can understand the reasoning behind the model’s predictions [33]. XAI techniques can shed light on the important factors the model is using to make its predictions, allowing clinicians to validate the model’s logic and build trust in the technology.

AI-based methodologies have the potential to improve prenatal diagnosis of birth defects and outcomes in assisted reproductive technology [32]. XAI can be used to explain the AI model’s decision-making process in these sensitive areas, enabling clinicians to better understand the rationale and have confidence in the model’s recommendations [34]. Additionally, real-time electronic health monitoring combined with XAI can help track maternal and fetal health during pregnancy, especially in low-resource settings.

### 2.3 Low birth weight detection

There has been extensive research conducted on classification and prediction of LBW using various classical machine learning algorithms such as K-Nearest Neighbors (KNN), Naive Bayes, Support Vector Machines (SVM), and Artificial Neural Networks (ANNs), as well as more advanced deep learning methods. Khan et al. [12] trained ten different classical machine learning algorithms were applied and discovered that Logistic Regression (LR) produced the best results for detecting LBW. The study used the synthetic minority oversampling technique (SMOTE) to address the class imbalance problem by increasing the minority class datapoints. Similarly, Feng et al. [35] applied SVM for LBW and high birth weight classification. SMOTE was used to handle the imbalance class problem. The study by Kuhle et al. [36] compared the performance of LR with other classical machine learning models and concluded that other models do not offer any significant improvement over LR. They also applied SMOTE to improve the representation of minority points, addressing the issue of class imbalance. Similarly, the Ren et al. [6] conducted extensive experimentation using seven different classical ML models i.e., LR, Naive Bayes, RF, Extreme Gradient Boosting (XGBoost), Adaptive Boosting, Multilayer Perceptron (MLP), and ANN along with four different data rebalancing methods such as random undersampling, random oversampling, SMOTE, and weight rebalancing, to address the class imbalance problem. The results indicated that XGBoost with weight rebalancing yielded the best performance.

In a study of Akhtar et al. [37] on birth weight classification, SVM, LR, Naive Bayes, and RF were applied for classification, with SVM yielding the best results. To handle the class imbalance issue, a data-based ensemble strategy was proposed. The proposed technique involved dividing a dataset with 189,342 controls and 26,226 cases of LBW into two sets. One for LBW and other for non-LBW. The non-LBW set was further partitioned into 10 equal parts, each merged with the LBW records to create 10 new balanced datasets. The created datasets were then used to train and evaluate the performance of classification algorithms to ensure that each model is tested against a balanced representation of both classes.

Based on the discussed articles, we find that most of the literature treats the detection of LBW as a binary classification problem and addresses the class imbalance issue using oversampling and undersampling techniques. However, undersampling the majority class data points can cause a significant loss of information [38], while oversampling the minority class data points can lead to overfitting and changing of the decision boundary [39].

To the best of our knowledge, this is the first experimentation to compare and analyze unsupervised state-of-the-art anomaly detection algorithms for low, very low and extreme birth weight detection. In addition to providing computational efficient interpretability about the applied anomaly detection models for each detection problem.

## 3 Proposed methodology

**Fig 1:**
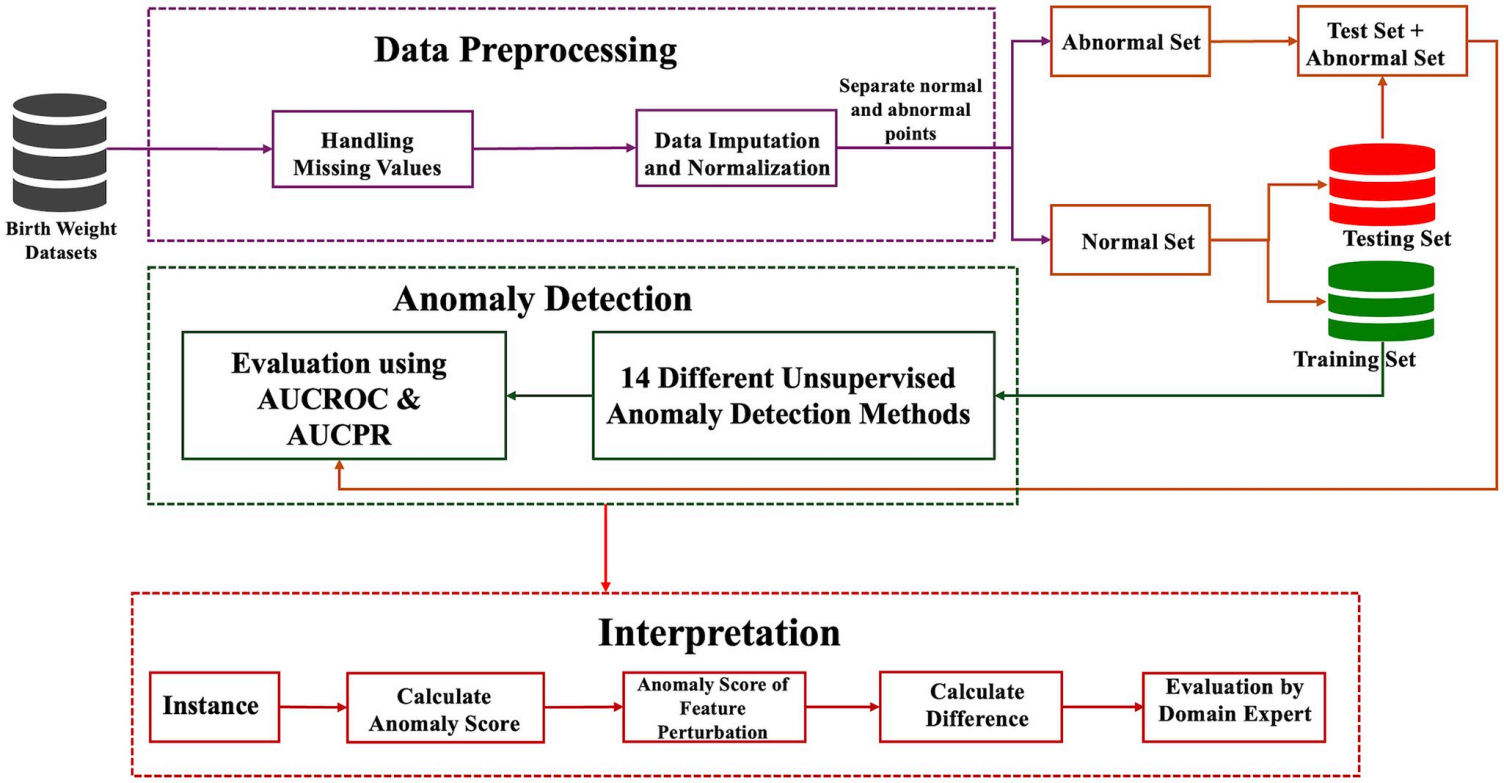
Schematic diagram illustrating the proposed approach for LBW detection.

The proposed methodology is presented in Fig 1. and divided into three steps: 1) Data collection and preprocessing; 2) Anomaly detection; 3) Feature perturbation interpretation. Intitially, the datasets is collected by UAE University under the umbrella of Mutaba’ah study [40] which consists of 3509 instances. The dataset undergoes through rigorous preprocessing to address missing values, normalize numerical data, and encode categorical variables, ensuring its understandable for analytical models. Then a diversified set of fifteen anomaly detection models were applied to performed anomaly detection on different segments of the data which represents low, very low, and extreme birth weight categories. Each model was carefully chosen to cover a wide range of anomaly detection approaches, with performance measured using Area Under the Receiver Operating Characteristics (AUCROC) and Area Under the Precision-Recall Curve (AUCPR) measures [41]. The approach not only facilitated in the identification of normal and anomalous instances within each birth weight category but it also allowed for the comparison of model effectiveness across different datasets. Finally, a feature perturbation technique was applied for interpreting the outcomes of anomaly detection models. By systematically changing specific features and observing the resulting changes in an anomaly score of instances, the proposed methodology revealed insights into the relative importance of numerous prenatal features influencing individual instance of birth weight. The interpretative analysis has important implications for early intervention methods for birth weight detection.

**Algorithm 1** Proposed Algorithm

1: **Input:** Dataset **X**, labels **y**

2: **Output:** Average AUCROC and Average AUCPR, list of features sorted by impact

3: **procedure** Preprocessing

4:  Load dataset **X** and labels **y**

5:  Categorize ‘weight’ into new categories:

6:  low_birth_weight = *weight* < 2500

7:  very_low_birth_weight = *weight* < 1500

8:  extreme_low_birth_weight = *weight* < 1000

9:  Convert ‘weight’ categories to categorical variable *lbw*

10:  **for** each numerical column in **X** excluding ‘lbw’ **do**

11:   Impute missing values using the mean of the column

12:  **end for**

13:  **for** each categorical/binary column in **X** **do**

14:   Impute missing values using the mode of the column

15:   **if** binary **then**

16:    Convert ‘Yes’/‘No’ to 1/0

17:   **end if**

18:   Convert categorical data to numerical using one-hot encoding

19:  **end for**

20:  Normalize/Standardize the numerical columns of **X**

21: **end procedure**

22: **procedure** Anomaly Detection

23:  Train Anomaly detection model on the normal training subset of **X**

24:  **for** each fold in 2x5-fold cross-validation **do**

25:   Ensure both classes are present in the test subset

26:   Apply Anomaly detection models to the test subset and compute scores

27:   Calculate AUCROC and AUCPR

28:  **end for**

29:  Compute average AUCROC and AUCPR across all folds

30: **end procedure**

31: **procedure** Feature Importance with Anomaly Detection Models

32:  For a given test instance and list of features:

33:  Calculate the original anomaly score

34:  **for** each feature **do**

35:   Replace feature value with the mean

36:   Recalculate anomaly score

37:   Store the difference in scores

38:  **end for**

39:  Sort features by the absolute value of their score differences

40: **end procedure**

### 3.1 Data collection and preprocessing

#### 3.1.1 Ethics statement

The dataset is part of the Mutaba’ah study which is the regionally pioneering longitudinal cohort investigation focusing on mother and child health dynamics [42]. The dataset is collected in Al Ain, Abu Dhabi, UAE. Specifically, pregnant women from the Emirati population were recruited and monitored through hospital medical records. Ethical approval for the study was granted by the Abu Dhabi Health Research and Technology Ethics Committee (DOH/CVDC/2022/72), adhering strictly to the Declaration of Helsinki guidelines. The data presented on this study can be made available on request from the Mutaba’ah Study. Approval from a research ethics comitee may be required. Request can be sent to the Mutaba’ah study PI (luai.ahmed@uaeu.ac.ae).

#### 3.1.2 Datasets

The resultant dataset comprises 3509 instances, each characterized by 42 unique features. However, our method focus narrows down to select a 22 subset of features related to the first trimester of pregnancy which is aligning with the WHO classifications for LBW [1]. The detailed categorization divides LBW into three distinct categories: low, very low, and extreme LBW. Our analytical framework, therefore, dissects these categories to detect birth weight with increased specificity and accuracy by tailoring the analysis to early pregnancy stages.

- **Dataset Overview:** A total of 3509 instances, with 22 distinct features focusing on first trimester only.
- **Problem Stratification:** Analysis segmented according to WHO birth weight categories—low, very low, and extreme LBW.
- **Objective:** To enhance detection accuracy and interpretation in early pregnancy birth weight detection.

The datasets used in our experiments contained ambiguities such as noise and missing values, so we applied various preprocessing techniques to prepare the data for further analysis. The preprocessing techniques are explained below;

#### 3.1.3 Handling missing values

Two different and simple methods were applied for imputation to handle missing values in both numerical and categorical features. The mean value of the corresponding feature throughout the dataset was used to fill in any missing values for numerical features which will guarantees that the scale and general distribution of the numerical data are unchanged [43]. On the other hand, the most prevalent category within each feature was preserved for categorical features by using the mode of each feature to fill in any missing values [44].

#### 3.1.4 Feature transformation and normalization

All categorical features were transformed to numerical values in order to make computational analysis more easier. Then min-max normalization was applied to the numerical features [45]. By rescaling the data to a constant range of 0 to 1, this method reduces the probability of bias caused by variables with varying scales and enables consistent comparison across features.

Datasets is inherently imbalance therefore we choose to handle this problem as anomaly detection problem. Specifically, the dataset was segmented into minority or anomalous and majority or normal classes based on the birth weight categories established for the experimentation [46]. For the purpose of training anomaly detection models, only instances classified as normal were utilized by following the foundational principle of anomaly detection models where the focus is on learning from a single normal class.

### 3.2 Anomaly detection (AD)

Anomaly detection models are an essential tool in identifying patterns in data that differ significantly from the normal. These models are designed to detect outliers or abnormalities using a range of statistical, machine learning, and computational methods [47]. The output of anomaly detection models are outlier score or anomaly score [48]. An anomaly score quantifies the degree of deviation of a data point from the expected pattern; a higher score indicates a higher likelihood of the point being an anomaly. In the context of LBW detection, anomaly detection models excel at identifying cases that deviate from normal birth weight ranges. The capability is invaluable for early identification of at-risk pregnancies, enabling timely and targeted interventions. By accurately discriminating between normal and abnormal cases [49]. The anomaly score enables clinicians and healthcare providers to assess the risk level of each case, prioritizing care and resources where they are most needed.

In the context of prenatal healthcare, the task of anomaly detection is pivotal, particularly when conducted in an unsupervised manner without explicitly labeled training data for potential deviations in birth weights to handle imbalance class problem [50]. Our experimentation focuses on various anomaly detection models designed to differentiate between normal and abnormal instances of birth weights, categorized into low, very low, and extreme LBW. We employed fourteen different AD as summarized in Table 1 for each category separately.

**Table 1:** Comparison of anomaly detection models.

Model	Definition	Key Parameters	Advantages	Disadvantages
ABOD [51]	Considers angles between difference vectors for outlier detection.	n_neighbors: number of neighbors to consider.	Handles multi-dimensional data well; robust against noise.	Computationally heavy; performance degrades with dimensionality.
CBLOF [52]	Integrates clustering with LOF for anomaly detection.	Number of clusters, size thresholds for small clusters.	Leverages cluster analysis; effective in diverse datasets.	Depends on clustering quality; less effective without clear cluster structure.
ECOD [53]	Uses empirical distribution functions for detecting outliers.	Number of bins for histogramming, distance metric.	No assumption about data distribution; effective in large datasets.	Choice of bins affects performance; may not capture multi-modal distributions.
GMM [54]	Models data with a mixture of Gaussian distributions to identify outliers.	n_components: number of Gaussian distributions, covariance type.	Models complex distributions well; provides soft clustering.	Sensitive to initialization; may struggle with high-dimensionality.
HBOS [55]	Assumes feature independence and computes outlier score based on histograms.	Number of bins, static or dynamic bin width.	Fast and scalable; suitable for high-dimensional data.	Assumes independence among features; may overlook relationships.
Isolation Forest [56]	Isolates anomalies using trees.	n_estimators: number of trees, max_samples: size of sample, contamination: proportion of outliers.	Efficient in high dimensions; handles outliers well.	Random selection may cause variability; not best for small datasets.
KDE [57]	Estimates probability density function to find outliers.	Bandwidth: controls the smoothness of the estimate.	Flexible modeling of distributions; effective in high-dimensional space.	Bandwidth selection critical; computationally expensive.
KNN [58]	Uses distance to the kth nearest neighbor as the outlier score.	k: number of neighbors.	Simple and intuitive; effective with defined neighborhoods.	Computationally demanding; sensitive to k.
LODA [59]	Employs random projections and histograms for anomaly detection.	Number of random cuts, number of bins.	Efficient for online and large datasets; low memory footprint.	Variable performance with parameter choices; less effective for complex patterns.
LOF [60]	Measures local density deviation with respect to neighbors.	n_neighbors: number of neighbors to consider.	Identifies local outliers effectively; adaptable to clusters of varying density.	Computationally intensive; sensitive to n_neighbors.
One-Class SVM [61]	SVM variant that creates a boundary around data points.	Kernel: type, nu: margin errors, gamma: kernel coefficient.	Effective in non-linear pattern recognition; works in high-dimensional space.	High computational cost; sensitive to tuning.
PCA [62]	Uses PCA for dimensionality reduction and identifies anomalies based on reconstruction error.	n_components: number of principal components.	Reduces dimensionality; efficient in reduced space.	Assumes linear relationships; sensitive to n_components.
SOS [63]	Evaluate the likelihood of each data point being an outlier based on its relationship with neighboring points.	Affinity Matrix, Binding Probability Matrix	Effective in diverse scenarios	Sensitive to parameter settings and requires careful interpretation.
SUOD [64]	Accelerates heterogeneous outlier detection by optimizing base detectors.	Base detectors, computation budget, parallelization strategy.	Scalable; flexible with choice of base detectors.	Complex configuration; reliant on base detector performance.

The dataset division aligns instances with their respective birth weight categories as interpreted in Table 2, further splited into training and testing sets. Each AD model undergoes meticulous hyperparameter optimization to ensure suitability to the characteristics of dataset.

**Table 2:** Categorization of used birth weights datasets.

Categories	Total Samples	Normal	Abnormal	Default Ratio
LBW	3509	3099	410	0.116
Very LBW	3509	3464	45	0.0128
Extreme LBW	3509	3490	19	0.0054

The effectiveness of AD models is inherently dependent on the specification of dataset. Our exploration spanned a range of models, from the IF, effective in isolating outliers within high-dimensional data, to the ECOD model, which operates effectively across vast datasets without presuming data distribution.

The core of our approach is the anomaly score computation:

$$\begin{array}{c} S(x)=\phi (x) \end{array}$$ (2)

where *S*(*x*) represents the anomaly score for instance *x*, and *ϕ*(*x*) denotes the decision function of the AD model. A higher score indicates a significant deviation from normalcy.

The exploration offers a scalable, adaptable anomaly detection framework in prenatal health, demonstrating capability of unsupervised learning to illuminate birth weight variations. By diligently applying and assessing a variety of AD models, we distinguish complexities of prenatal health indicators, paving the way for preemptive measures and enhanced comprehension.

### 3.3 Feature perturbation interpretation

The technique involves modifying a single feature of the instance at one iteration and observing the variation in the anomaly score. The modification typically replaces the original value of feature with a statistical measure mean in this case derived from normal instances in the training set. Algorithm 2 provides the explanation of anomaly score perturbation interpretation method,

In Algorithm 2, *x*[*j*] is the *j*^*th*^ feature of *x*, and *μ*_*i*_ represents the mean value of the *i*^*th*^ feature across the instances in the testing set, denoted as *X*_test,instance_. Consequently, the anomaly score of the modified instance is $S_{m,i}=f\left(x_{i}^{\prime }\right)$.

The change in anomaly score attributable to the *i*^*th*^ feature, denoted as Δ*S*_*i*_, is then calculated as:

$$\Delta S_{i}=S_{o}-S_{m,i}$$

A positive Δ*S*_*i*_ signifies that the feature’s original value contributes towards making the instance appear less anomalous (i.e., more normal), while a negative Δ*S*_*i*_ indicates that the feature exacerbates the instance’s anomaly.

**Algorithm 2** Feature Perturbation Interpretation Algorithm

1: **Input:** Instance $x\in R^{d}$ with *d* features, anomaly detection model *f*, mean values *μ*_*i*_ for each feature *i* calculated from normal instances in the training set

2: **Output:** Feature importance values Δ*S*_*i*_ for each feature *i*

3: **procedure** Feature Perturbation Interpretation

4:  Calculate the original anomaly score of **x**: *S*_*o*_ = *f*(**x**)

5:  **for** each feature *i* in {1, 2, …, *d*} **do**

6:   Define a modified instance $x_{i}^{\prime }$ where:

$$x_{i}^{\prime }\left[j\right]=\{\begin{array}{cc} x[j], & \text{if}\;j\neq i\\ \mu_{i}, & \text{if}\;j=i \end{array}$$

7:   Calculate the anomaly score of the modified instance: $S_{m,i}=f\left(x_{i}^{\prime }\right)$

8:   Compute the change in anomaly score attributable to the *i*th feature: Δ*S*_*i*_ = *S*_*o*_ − *S*_*m*,*i*_

9:  **end for**

10:  Sort features by the absolute value of their score differences Δ*S*_*i*_

11: **end procedure**

12: **procedure** Interpretation

13:  **for** each feature *i* **do**

14:   **if** Δ*S*_*i*_ > 0 **then**

15:    The original value of feature *i* makes the instance appear less anomalous (i.e., more normal)

16:   **else if** Δ*S*_*i*_ < 0 **then**

17:    The original value of feature *i* increases the instance’s anomaly score

18:   **end if**

19:  **end for**

20: **end procedure**

The proposed method directly quantifies feature significance in anomaly detection without requiring the extensive computations of techniques like LIME and SHAP by making it efficient and practical for clinical use. By identifying the prenatal features that most significantly affect LBW predictions, this approach increases transparency and provides clinicians with actionable insights.

Conventional methods such as SHAP and LIME provide interpretability by approximating model predictions or evaluating feature importance. However, they often require significant computational resources. Moreover, these methods encounter challenges in accurately explaining outlier behavior in unsupervised scenarios, particularly when working with highly imbalanced data.

## 4 Results and discussion

In this section, first we present the results obtained from the experimentation then results are discussed.

### 4.1 Experimental setup

For our experimentation on understanding birth weight categories using anomaly detection, we used advance technology and tools to set up an experiment. The PyOD library was selected to run our models for anomaly detection [65]. PyOD is a recommended library for this type of work due to its abundance of various techniques for identifying outliers in data. It integrates well with other Python tools we use, such as pandas and NumPy, to improve the efficiency and flow of our data processing. We utilized a PC equipped with a 16 GB RAM NVIDIA GeForce RTX 4090 graphics card.

### 4.2 Evaluation metrics

We employed two commonly used metrics, AUCROC and AUCPR to assess the effectiveness of anomaly detection models specifically helpful for testing models on imbalanced datasets [41]. A capacity of model to differentiate between classes is measured by its AUCROC, where a higher AUC denotes better model performance. An AUCROC value greater than 0.5 reveals that the model has a better than random chance in distinguishing between the classes with values closer to 1.0 indicating best classification abilities. However, when working with severely imbalanced data, the AUCPR is seen to be more useful than ROC because it concentrates on the precision-recall trade-off. High accuracy (a low false positive rate) and high recall (a low false negative rate) are indicative of a higher AUCPR score, both of which are critical for medical diagnosis. For AUCPR, a score higher than the proportion of minority classes out of the total sample set indicates that the model is effectively identifying the minority class instances. As the interpretation of the anomaly detection models is crucial for clinical applications therefore the evaluation of interpretation part was conducted with the help of domain experts specifically clinicians knowledgeable about prenatal health. The expertise of clinicians ensured the relevancy and effectiveness of our findings as they could contextualize the anomaly detection and interpretation results within the broader spectrum of maternal and fetal health.

Additionally, we also measured the processing time needed for anomaly detection and interpretation to demonstrate the effectiveness of our method. Less computing times signify an enhanced efficiency of the method by implying its practicality in real-life scenarios where prompt decision-making can be pivotal. The involvement of domain experts not only enhanced the validity of our interpretations but also demonstrated the potential of model for implementation in clinical settings.

### 4.3 Anomaly detection results

The results obtained by applying anomaly detection models mentioned in Section 2.1 are presented in this subsection.

The Table 3 reveals the results of LBW detection. The OCSVM model generates a better AUCROC which indicates that it is better in detecting abnormal LBW. On the other hand, models with lower AUCROC values indicating less reliability such as PCA and CBLOF. Models differ greatly in how long they took to perform computation for instance method like ABOD and SOS took a long time while other models like OCSVM and IF work faster.

**Table 3:** Comparison of LBW detection results.

Method	AUCROC	AUCPR	Computational Time (seconds)
IF	0.5434	0.1462	0.48
**OCSVM**	**0.57**	**0.167**	0.15
GMM	0.567	0.166	1.37
LODA	0.529	0.137	0.49
LOF	0.556	0.156	0.55
PCA	0.48	0.128	0.13
SUOD	0.54	0.149	33.4
KDE	0.524	0.154	0.62
KNN	0.534	0.154	0.61
ECOD	0.565	0.158	0.4
SOS	0.563	0.161	16.23
ABOD	0.528	0.15	6.01
CBLOF	0.489	0.13	1.02
HBOS	0.541	0.159	0.54

Similarly, in Table 4, the performance of various anomaly detection methods for identifying very LBW instances is assessed. The OCSVM exhibits the highest AUCROC score of 0.6424. On the other end, the CBLOF shows the least effective AUCROC score of 0.477 by suggesting that it may not be as effective in identifying very LBW cases in this context. When evaluating the balance between precision and recall with AUCPR, ECOD outperforms other models with a score of 0.045, which is significant given the class imbalance inherent in the very LBW detection task. In contrast, CBLOF records the lowest AUCPR score of 0.016. These observations highlights the importance of choosing the right balance between detection performance and computational efficiency with OCSVM standing out as strong choice in both respects for the detection of very LBW cases. Finally, Table 5 presents the anomaly detection results for extreme LBW instances. The OCSVM model leads the AUCROC with a score of 0.72. This is closely followed by the HBOS method, which achieves an AUCROC of 0.712. SOS excels in the AUCPR metric with 0.037, indicating its precision and recall balance is well-suited for this imbalanced data problem. In contrast, the CBLOF falls short in both AUCROC and AUCPR metrics with score of 0.405 and 0.009, respectively. These findings highlight that while OCSVM and HBOS show high effectiveness in anomaly detection for extreme LBW, PCA offers a time-efficient alternative. The results imply a trade-off between accuracy and efficiency, with OCSVM and HBOS providing higher detection rates and PCA offering faster processing times.

**Table 4:** Comparison of Very LBW detection results.

Method	AUCROC	AUCPR	Computational Time (seconds)
IF	0.634	0.0279	0.47
OCSVM	0.6424	0.0255	0.44
GMM	0.581	0.0289	1.54
LODA	0.59	0.018	0.32
LOF	0.629	0.0206	0.32
PCA	0.47	0.034	0.11
SUOD	0.5949	0.0224	33.54
KDE	0.56	0.0254	0.52
KNN	0.556	0.0301	0.44
**ECOD**	**0.653**	**0.045**	0.37
SOS	0.633	0.0246	16.23
ABOD	0.592	0.0254	5.93
CBLOF	0.477	0.016	0.97
HBOS	0.632	0.045	0.08

**Table 5:** Comparison of Extreme LBW detection results.

Method	AUCROC	AUCPR	Computational Time (seconds)
IF	0.69	0.025	0.46
**OCSVM**	**0.72**	0.0253	0.37
GMM	0.6412	0.017	1.43
LODA	0.507	0.0139	0.31
LOF	0.625	0.012	0.48
PCA	0.65	0.014	0.07
SUOD	0.6939	0.0301	27.12
KDE	0.66	0.0229	0.53
KNN	0.697	0.0238	0.58
ECOD	0.701	0.0221	0.18
SOS	0.703	**0.037**	14.6
ABOD	0.6957	0.0205	4.71
CBLOF	0.405	0.009	0.83
HBOS	0.712	0.0216	0.55

After the careful evaluation, an interesting observation emerges where the anomaly detection models seem to perform better as the the severity of low birth weight decreases moving from extreme to very low and then to general low birth weight, the data points begin to approximate the normal range more closely. This proximity reduces the distinctiveness of the anomalies, resulting in a higher likelihood of misclassification [66]. Therefore, the observed degradation in model performance with less extreme cases is expected and highlights the challenge of detecting anomalies that are less pronounced. This observation confirms the importance of developing detection methods that are sensitive enough to differentiate between near-normal and slightly abnormal cases, which are inherently more difficult to identify due to their subtle differences from the normal.

### 4.4 Statistical testing

The Table 6 summarizes the results of F-tests applied to the AUROC scores across birth weight categorizations. For LBW, the F-statistic is 2.353 with a significant p-value of 0.00759, indicating a statistically significant difference in model performances. For Very LBW, the p-value is 0.17799, suggesting no significant difference in performances. Finally, for Extreme LBW, the F-statistic is 0.425 with a non-significant p-value of 0.95763 which clearly indicating consistent model performance across evaluations.

**Table 6:** F-test on the AUCROC scores.

Birth Categorization	F-statistic	P-Value
Low Birth Weight	2.353	0.00759
Very Low Birth Weight	1.380	0.17799
Extreme Low Birth Weight	0.425	0.95763

Table 7 presents F-test results on the AUCPR scores for different birth weight categories. The F-statistics are relatively low across all categories, indicating no significant variance in model performances. P-values with 0.48591 are also high for LBW, 0.72941 for Very LBW, and 0.50685 for Extreme LBW, suggesting that the differences in model performance are statistically insignificant across these categories.

**Table 7:** F-test on the AUCPR scores.

Birth Categorization	F-statistic	P-Value
Low Birth Weight	0.969	0.48591
Very Low Birth Weight	0.731	0.72941
Extreme Low Birth Weight	0.948	0.50685

### 4.5 Interpretation results

After applying a wide range of anomaly detection models, we performed feature interpretation by concentrating mainly on the best performer model for each category and IF model specifically. The purpose of selecting the IF model was to create a reference point for comparing various birth weight situations as local-DIFFI is an interpretation technique specifically designed for the IF model.

To further explore the interpretability of the model, we carefully chose examples that span the different ranges of birth weights for each category. Their results are provided in the S1 File. For examples, the instances that fall into the following ranges: 2500 to 1500 grams, 1000 to 1500 grams, and less than 1000 grams. With this deliberate choice, the wide range of neonatal health issues from LBWs to extremely LBWs was intended to be captured. The followed process not only highlight the inner workings of the model but also highlighted the feature contribution in the detection process.

**Fig 2:**
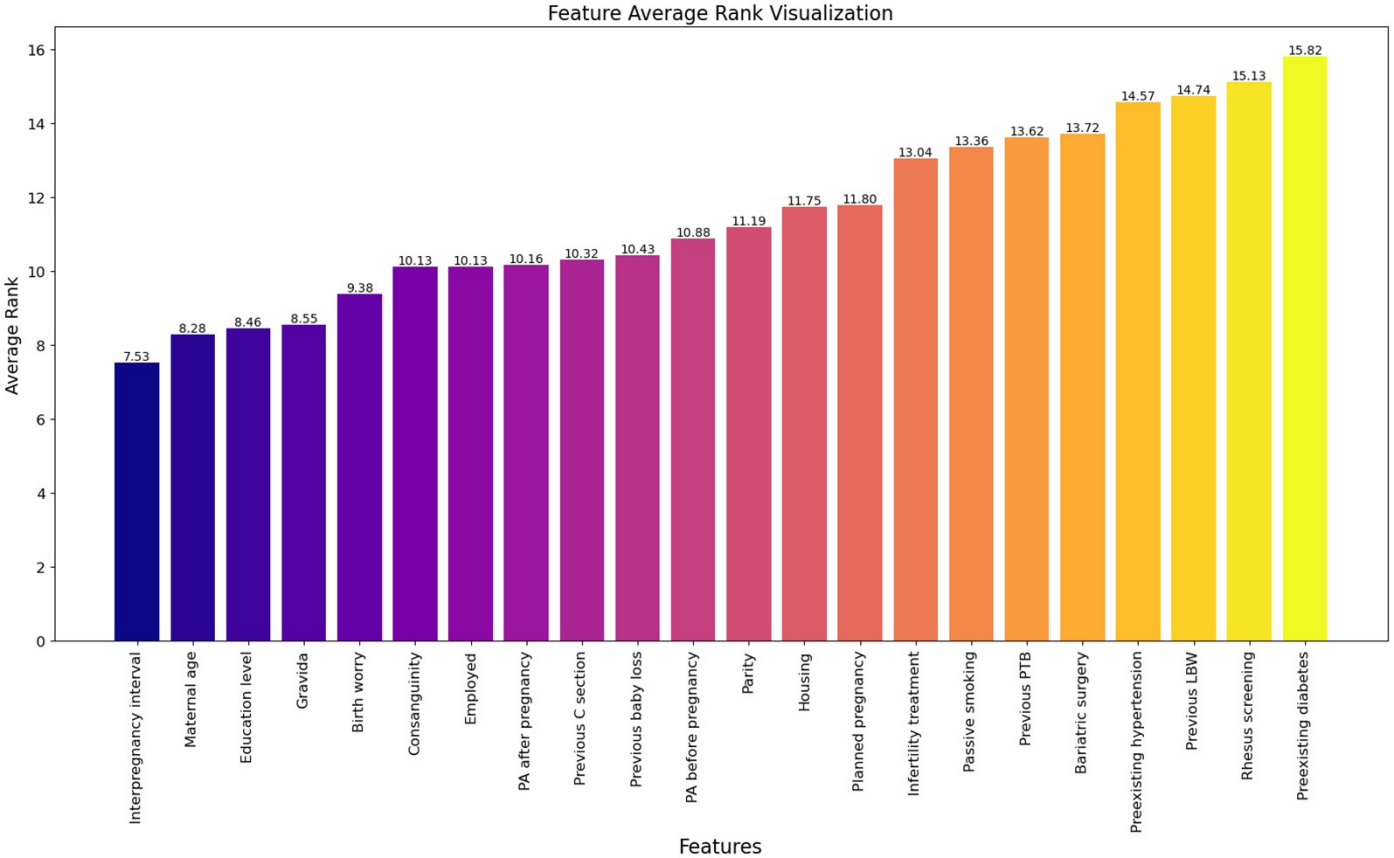
Average rank of features across outlier points for LBW using feature perturbation based interpretation.

**Fig 3:**
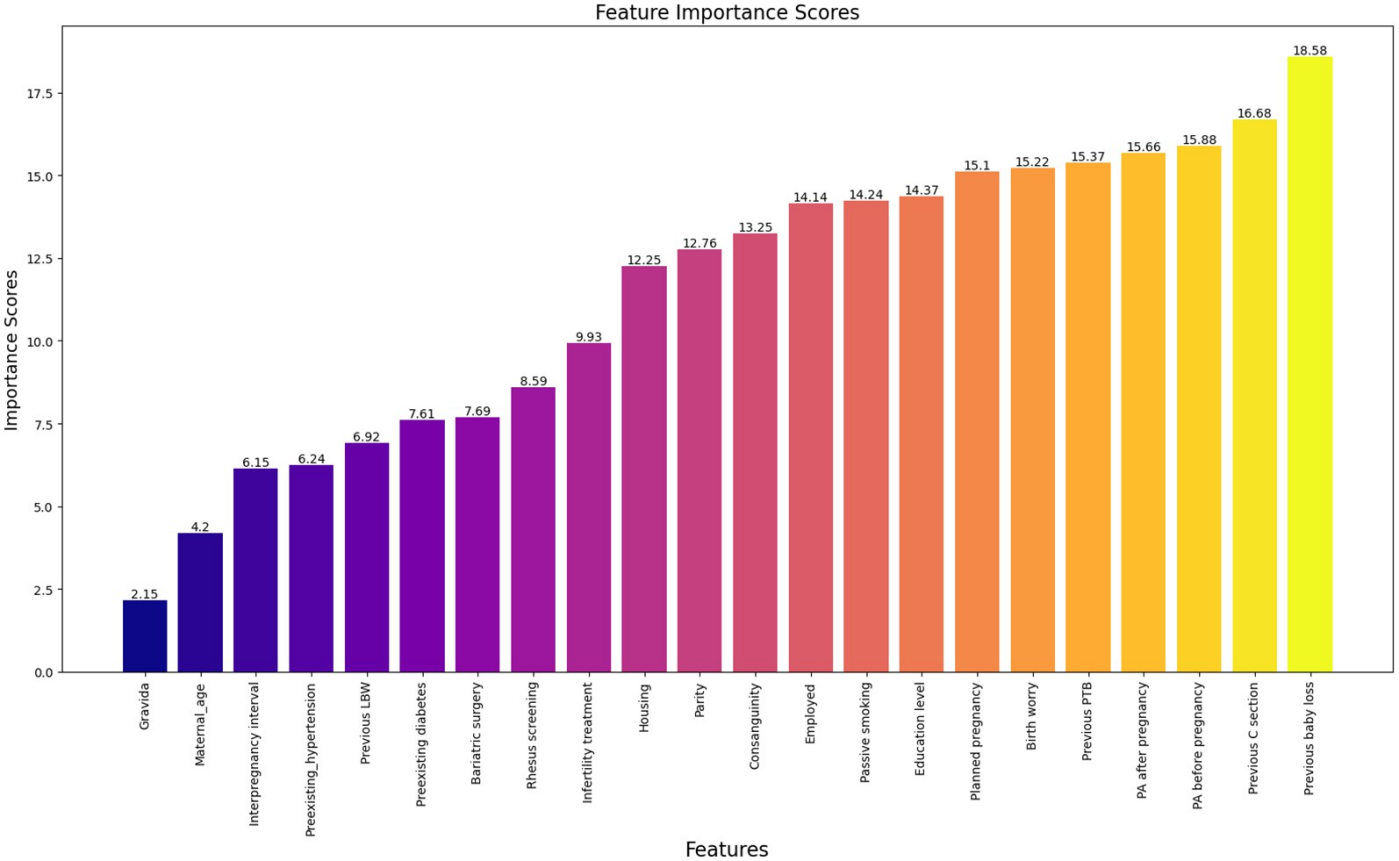
Average rank of features across outlier points for very LBW using feature perturbation based interpretation.

**Fig 4:**
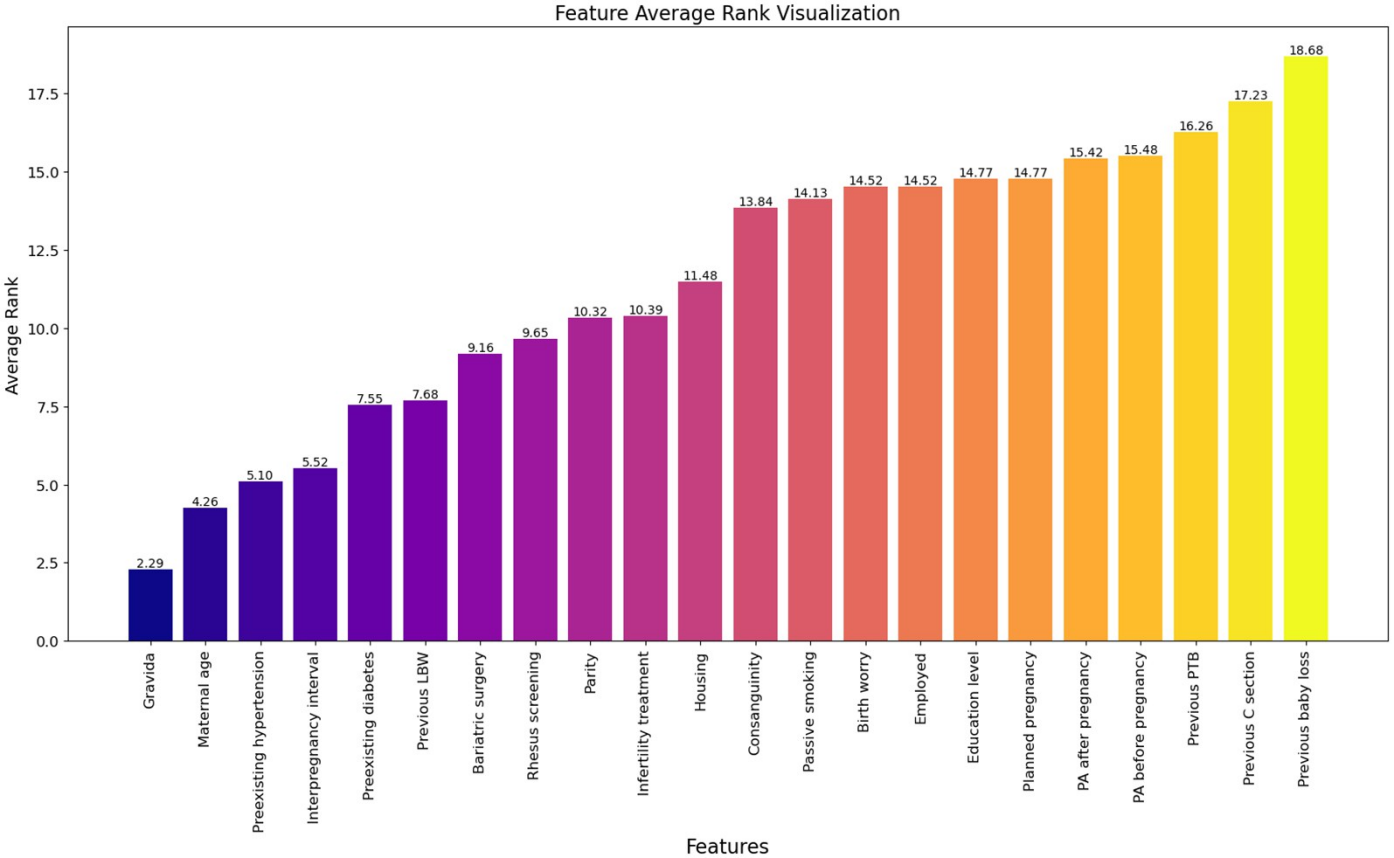
Average rank of features across outlier points for extreme LBW using feature perturbation based interpretation.

To provide a more detailed interpretation, we also calculated the average rank of all features across all outlier points for both interpretation methods. Figs 2–4 illustrate the feature perturbation interpretation of the average rank of all features across all outlier points for low, very low, and extreme low birth weights, respectively. Similarly, interpretations using local-DIFFI are provided in the S1 File.

The evaluation of proposed method is performed by domain experts. According to domain experts, the Local-DIFFI method is more interpretable and enhance the understanding from a clinical perspective. However, despite its more interpretability, the difference it presents when compared to our method is not significant additionally our method can be applied to any AD method while local-DIFFI is specific to IF only. The experimentation suggests that while Local-DIFFI facilitate a deeper understanding of the data from a domain-specific perspective, it does not substantially impact the overall analytical conclusions. Therefore, both methods provide valuable insights but further evaluation is needed to ascertain definitive advantages in clinical applications.

### 4.6 Clinical and scientific relevance

The proposed method focus on enhancing early detection and interpretation of different levels of birth weight by using AI and AD techniques. The proposed study highlights the maternal health risks associated with birth weight by emphasizing on the importance of early identification to mitigate potential health complications and long-term consequences such as increased morbidity, mortality, and chronic conditions in later stages. The research addresses the challenges of limited or unlabeled data typical in clinical settings by providing a method to detect pregnancies issues [67]. In urban hospitals with advanced neonatal care facilities, the proposed framework can be used to identify pregnancies that show risk of LBW, VLBW, and ELBW. The model could send an alert to doctors for further tests. Early identification of these issues allow clinicians to provide specific treatments such as need of more nutrition, bed rest, or monitoring for early labor which makes the neonatal care more precise. Scientifically, the proposed approach allows for preliminary insights which can guide further data labeling by ultimately aiming to improve survival rates, reduce healthcare costs, and enhance the quality of life for affected infants. The proposed feature perturbation technique further enhances the interpretability of the model and providing insights into the effect of various features on birth weight outcomes which ultimately contributes in evidence-based clinical decision-making.

### 4.7 Research implications

The proposed methodology provides a comprehensive framework for detecting and interpreting LBW using AD techniques. The approach not only helps in the early identification of LBW risks but also contributes to a deeper understanding of the factors influencing birth weights. Moreover, the introduction of an interpretation method enhances the transparency and usability of anomaly detection models [68], making them more accessible and reliable to clinicians and healthcare practitioners. This could lead to better-informed clinical decisions and potentially improve neonatal care strategies.

### 4.8 Strengths and limitations

The utilization of the AD techniques along with proposed interpretation technique enhances the reliability in birth weight detection. The application of diverse models provides a robust analysis of birth weight predictors by reinforcing the generalizability of the results across different settings [69].

However, there are certain limitations associated with proposed methodology. As the methodology focus on a UAE population which limit the applicability of the findings to other demographic areas with different socioeconomic and genetic factors [70]. Additionally, the reliance on self-reported data for few features introduce bias and datasets contain a large amount of missing values. While the feature perturbation technique provides valuable insights however, as it relies on replacing values with mean, which may not accurately represent the unique and complex variations that occur during each individual pregnancy. Although the method provides interpretable outputs, transforming these outputs into practical guidance use can be difficult for clinicians, specifically for those without experience in AI. Training sessions may be necessary for clinicians to understand the interpretation output in their decision-making processes. Despite these limitations, the applicability of the study into birth weight are valuable for advancing prenatal care practices.

## 5 Conclusion

A framework for early detection of LBW pregnancies using AD methods is proposed in this study. The framework addresses problems of imbalanced data and a limited availability of labeled samples. Handling LBW as an AD problem allows the early and accurate identification of high risk pregnancies by facilitating preventative medical interventions without the need for large labeled datasets. Additionally, the anomaly score perturbation method improves interpretability by providing healthcare professionals with important information on crucial neonatal risk factors. The proposed framework has demonstrated efficiency and adaptability that is similar to established techniques i.e., Local-DIFFI. While the proposed framework reveals promise results, future work would validate its applicability across diverse populations by integrating longitudinal data for dynamic risk assessment while exploring hybrid models that combine AD with supervised learning as more labeled data becomes available.

## Supporting information

S1 File Supplementary file. The file contain the feature interpretation of randomly selected instances for each category.
(DOCX)
