# Correction: An efficient interpretable framework for unsupervised low, very low and extreme birth weight detection

**DOI:** 10.1371/journal.pone.0326707

**Published:** 2025-06-18

**Authors:** Ali Nawaz, Amir Ahmad, Shehroz S. Khan, Mohammad Mehedy Masud, Nadirah Ghenimi, Luai A. Ahmed

There is an error in affiliation 2 for author Shehroz S. Khan. The correct affiliation 2 is: College of Engineering and Technology, American University of the Middle East, Egaila, 54200, Kuwait.
