# Supplementary material for: An efficient interpretable framework for unsupervised low, very low and extreme birth weight detection
Source: PLoS One. 2025 Jan 30;20(1):e0317843. doi: 10.1371/journal.pone.0317843 (PMC11781751; doi:10.1371/journal.pone.0317843)
Supplement: S1 File — The file contain the feature interpretation of randomly selected instances for each category. (DOCX) [file pone.0317843.s001.docx]

**Supplementary File**

Table 0. Variable used

| Bariatric surgery | History of bariatric surgery: (Yes/No) |
| --- | --- |
| Birth worry | Worry about upcoming childbirth: (Yes/No) |
| Consanguinity | Consanguinity: (Yes/No) |
| Education level | Education level: (High/Low) |
| Employed | Employment: (Yes/No) |
| Gravida | Gravida: (1, 2, 3, …) |
| Housing | Housing: (Owned/Rented) |
| Infertility treatment | Previous infertility treatment: (Yes/No) |
| Maternal age | Maternal age: (18, 19, 20, …) |
| PA before pregnancy | Physical activity before pregnancy: (Yes/No) |
| PA during pregnancy | Physical activity during pregnancy: (Yes/No) |
| Parity | Parity: (1, 2, 3, …) |
| Passive smoking | Passive smoking: (Yes/No) |
| Planned pregnancy | Planning status for this pregnancy: (Yes/No) |
| Pre-existing diabetes | Pre-existing diabetes mellitus: (Yes/No) |
| Pre-existing hypertension | Pre-existing hypertension: (Yes/No) |
| Previous baby Loss | Previous losses: (Yes/No) |
| Previous C-section | Previous C-sections: (Yes/No) |
| Previous PTB | Previous preterm births: (Yes/No) |
| Previous LBW | Previous low birthweight: (Yes/No) |
| Rhesus screening | Rhesus (Rh) screening: (Yes/No) |

**Interpreting the values greater than 2500**

Category: Normal, Instance Index: 3153, Weight: 2875 g

Instance Values:

PA during pregnancy 0.000000

PA during pregnancy 0.000000

Bariatric surgery 0.000000

Birth worry 1.000000

Consanguinity 0.000000

Education level 0.000000

Employed 0.000000

Housing 1.000000

Infertility treatment 0.000000

PA before pregnancy 0.000000

Parity 2.000000

Passive smoking 0.000000

Planned pregnancy 1.000000

Preexisting Diabetes 0.000000

Preexisting Diabetes 0.000000

Previous LBW 0.000000

Rhesus screening 1.000000

Previous C section 0.000000

Previous baby Loss 0.000000

Previous PTB 0.000000

MATERNAL AGE 0.441993

GRAVIDA 0.105263

Name: 3153, dtype: float64

**Interpreting the values between 1500 than 2500**

Category: Low, Instance Index: 2929, Weight: 2105 g

Instance Values:

PA during pregnancy 0.000000

Bariatric surgery 0.000000

Birth worry 1.000000

Consanguinity 0.000000

Education level 0.000000

Employed 0.000000

Housing 1.000000

Infertility treatment 1.000000

PA before pregnancy_ 1.000000

Parity 4.000000

Passive smoking 1.000000

Planned pregnancy 1.000000

Preexisting Diabetes 0.000000

Preexisting Diabetes 1.000000

Previous LBW 1.000000

Rhesus screening 1.000000

Previous C section 4.000000

Previous baby Loss 2.000000

Previous PTB 1.000000

MATERNAL AGE 0.759037

GRAVIDA 0.315789

Name: 2929, dtype: float64

**Interpreting the values between 1000 than 1500**

Category: Very Low, Instance Index: 501, Weight: 1060 g

Instance Values:

PA during pregnancy 0.000000

Bariatric surgery 0.000000

Birth worry 1.000000

Consanguinity 0.000000

Education level 1.000000

Employed 0.000000

Housing 1.000000

Infertility treatment 0.000000

PA before pregnancy 1.000000

Parity 0.000000

Passive smoking 0.000000

Planned pregnancy 0.000000

Preexisting Diabetes 0.000000

Preexisting Diabetes 0.000000

Previous LBW 0.000000

Rhesus screening 1.000000

Previous C section 0.000000

Previous baby Loss 0.000000

Previous PTB 3.000000

MATERNAL AGE 0.170357

GRAVIDA 0.000000

Name: 501, dtype: float64

**Interpreting the values between less than 500**

Category: Extreme, Instance Index: 2889, Weight: 465 g

Instance Values:

PA during pregnancy 0.000000

Bariatric surgery 0.000000

Birth worry 1.000000

Consanguinity 0.000000

Education level 1.000000

Employed 1.000000

Housing 0.000000

Infertility treatment 0.000000

PA before pregnancy 1.000000

Parity 4.000000

Passive smoking 1.000000

Planned pregnancy 0.000000

Preexisting Diabetes 0.000000

Preexisting Diabetes 0.000000

Previous LBW 1.000000

Rhesus screening 1.000000

Previous C section 0.000000

Previous baby Loss 0.000000

Previous PTB 3.000000

MATERNAL AGE 0.347879

GRAVIDA 0.210526

Name: 2889, dtype: float64

**Low Birth Weight Category**


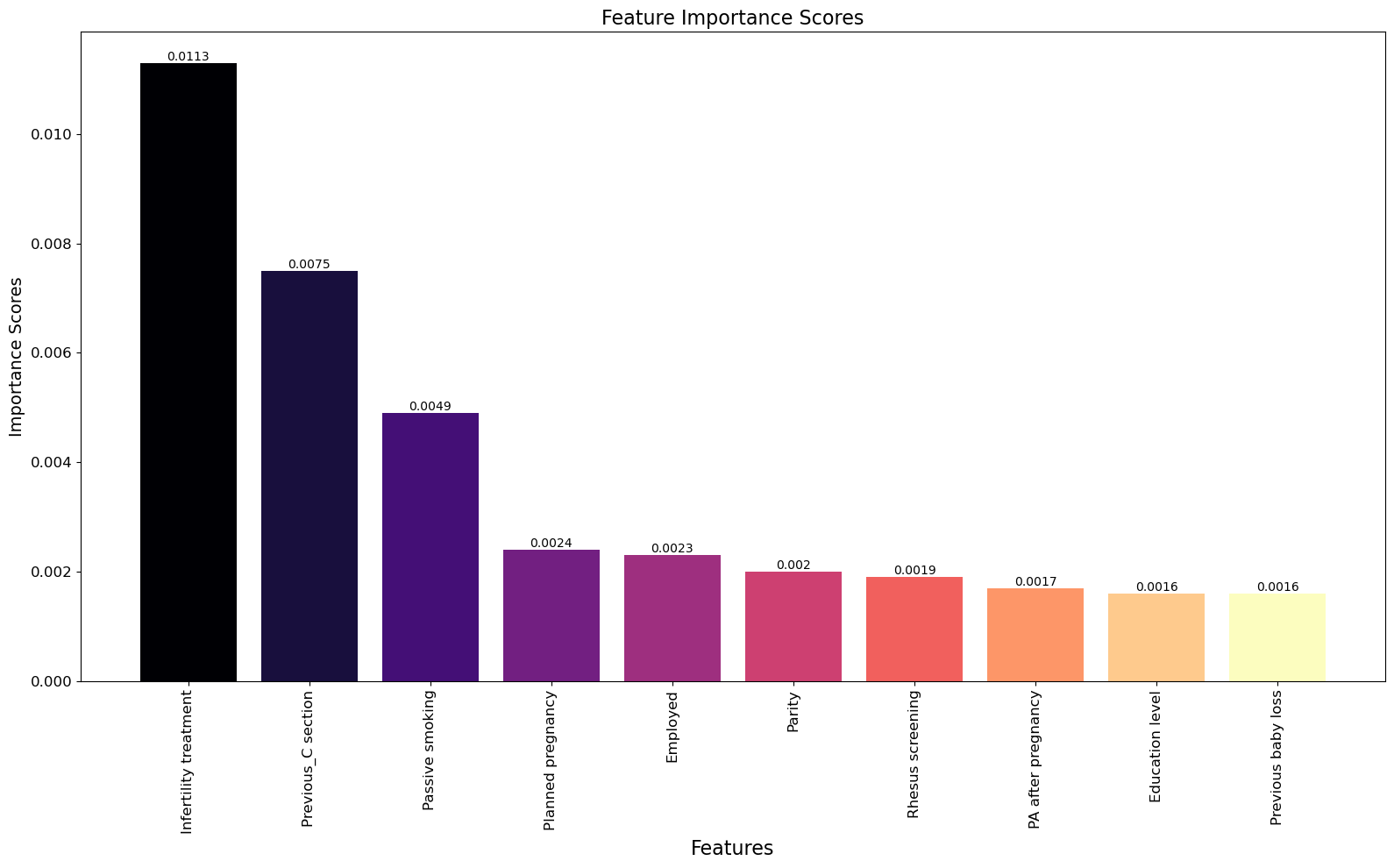


Figure 1. Feature contribution calculation by Local-DIFFI (weight=2895)

Table 1. Top 5 important features by Local-DIFFI (weight=2895)

| **Rank** | **Feature** | **Importance Score** |
| --- | --- | --- |
| 1 | Infertility treatment | 0.0123 |
| 2 | Previous C section | 0.0075 |
| 3 | Passive smoking | 0.0049 |
| 4 | Planned pregnancy | 0.0024 |
| 5 | Employed | 0.0023 |


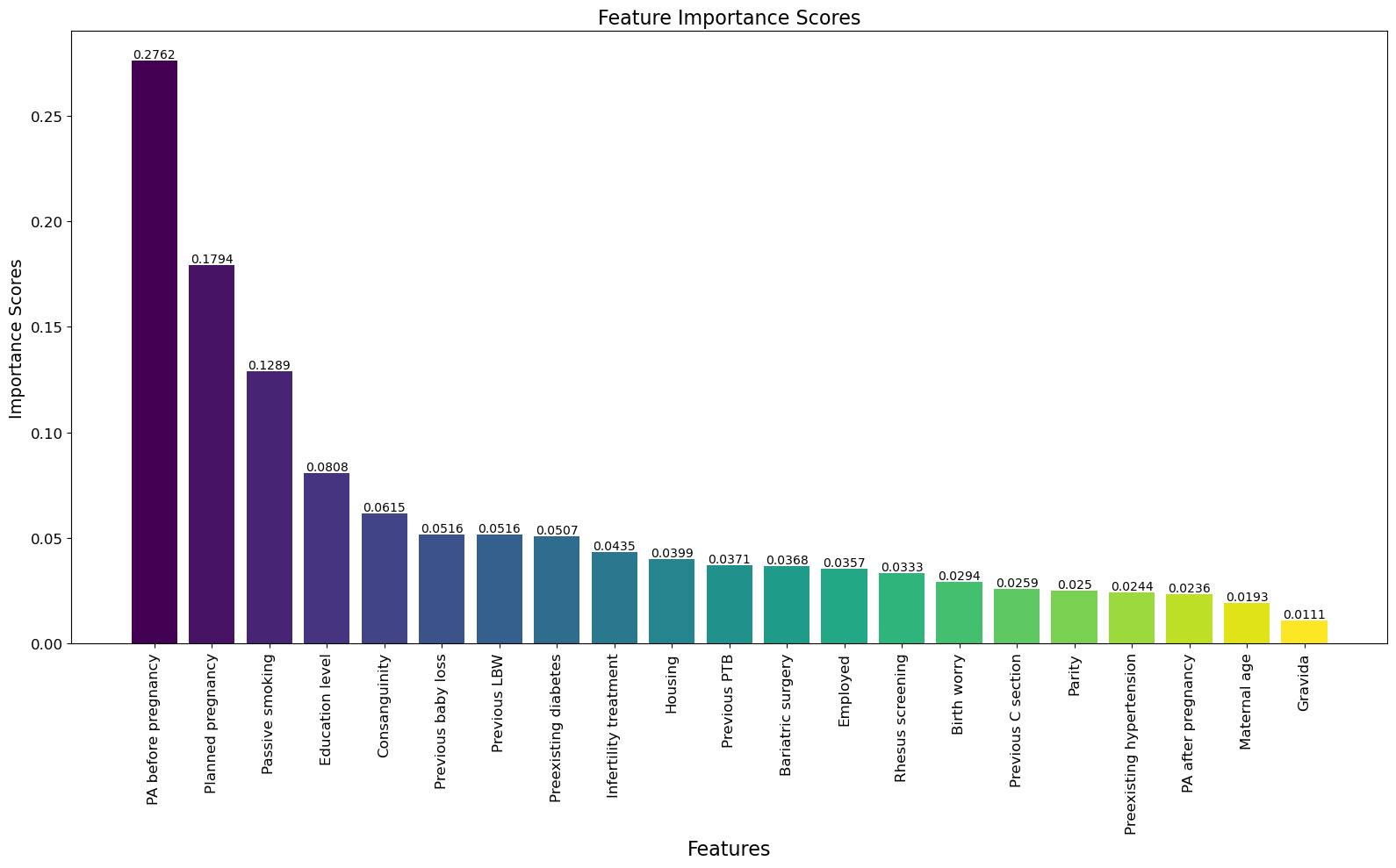


Figure 2. Feature contribution calculation by Local-DIFFI (weight=2105)

Table 2. Top 5 important features by Local-DIFFI (weight=2105)

| **Rank** | **Feature** | **Importance Score** |
| --- | --- | --- |
| 1 | PA before pregnancy | 0.2762 |
| 2 | Planned pregnancy | 0.1794 |
| 3 | Passive smoking | 0.1289 |
| 4 | Education level | 0.0808 |
| 5 | Consanguinity | 0.0615 |


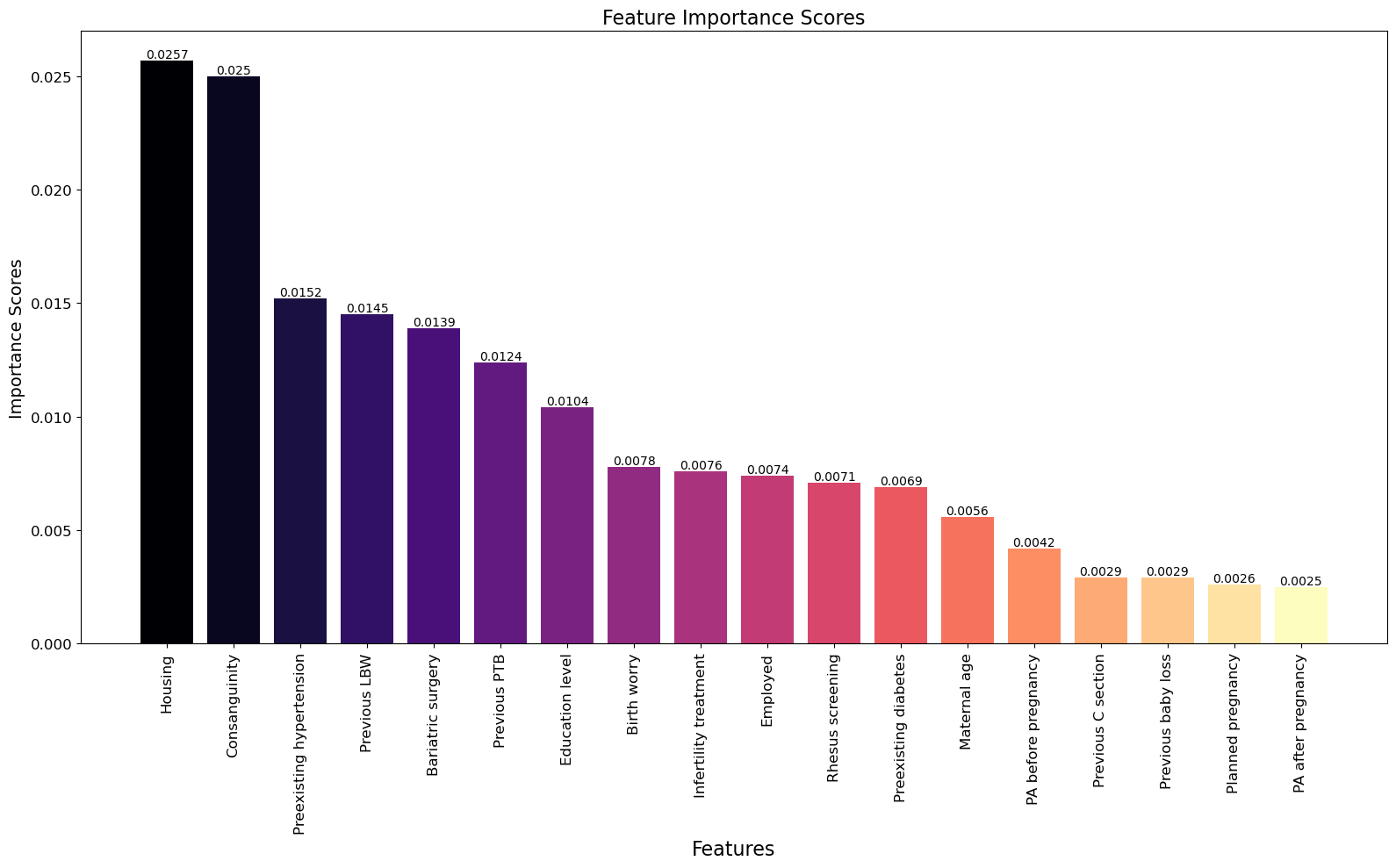


Figure 3. Feature contribution calculation by Local-DIFFI (weight=1060)

Table 3. Top 5 important features by Local-DIFFI (weight=1060)

| **Rank** | **Feature** | **Importance Score** |
| --- | --- | --- |
| 1 | Housing | 0.0257 |
| 2 | Consanguinity | 0.025 |
| 3 | Preexisting hypertension | 0.0152 |
| 4 | Previous LBW | 0.0145 |
| 5 | Bariatric surgery | 0.0139 |


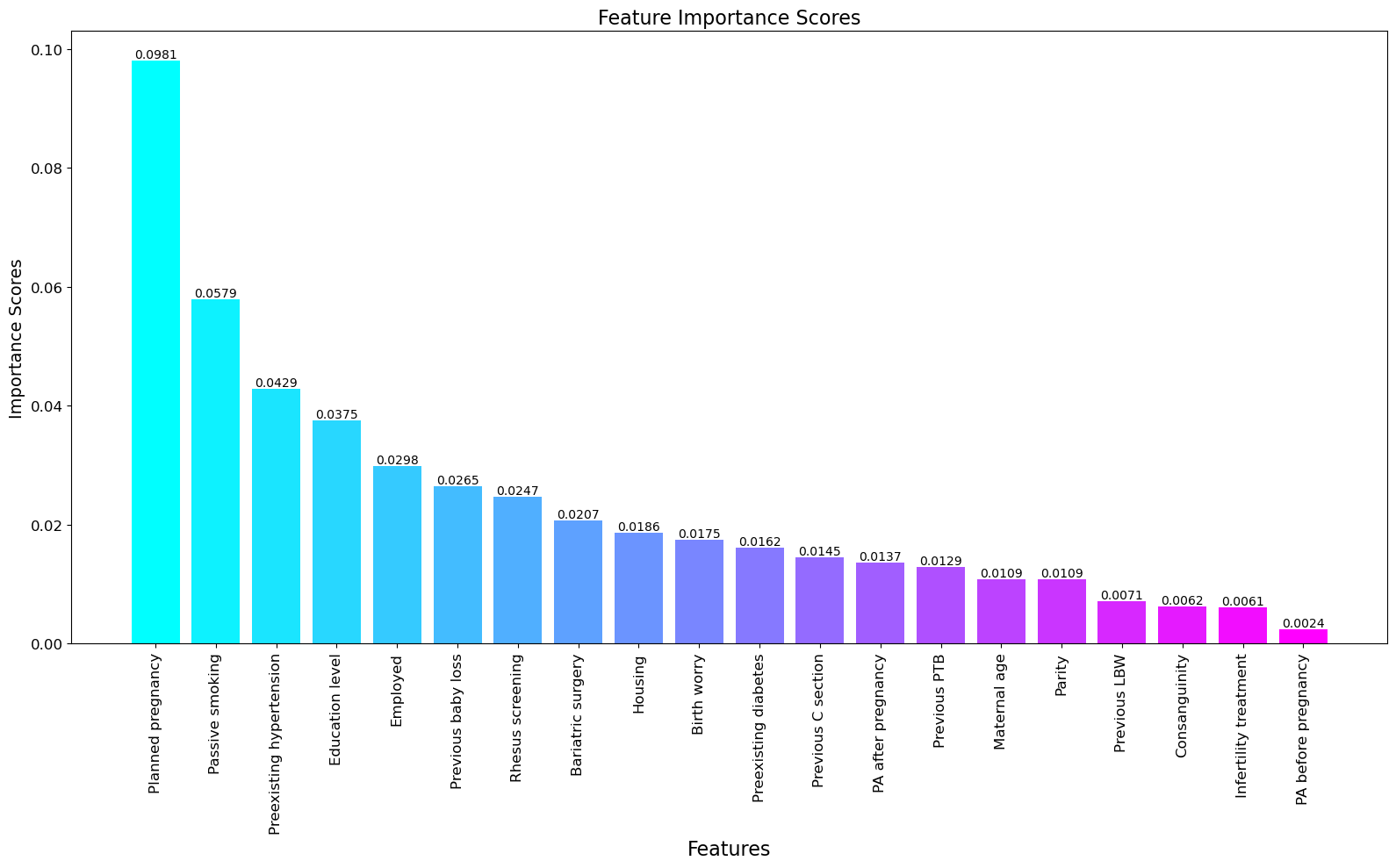


Figure 4. Feature contribution calculation by Local-DIFFI (weight=465)

Table 4. Top 5 important features by Local-DIFFI (weight=465)

| **Rank** | **Feature** | **Importance Score** |
| --- | --- | --- |
| 1 | Planned pregnancy | 0.0981 |
| 2 | Passive smoking | 0.0579 |
| 3 | Preexisting hypertension | 0.0429 |
| 4 | Education level | 0.0375 |
| 5 | Employed | 0.0298 |


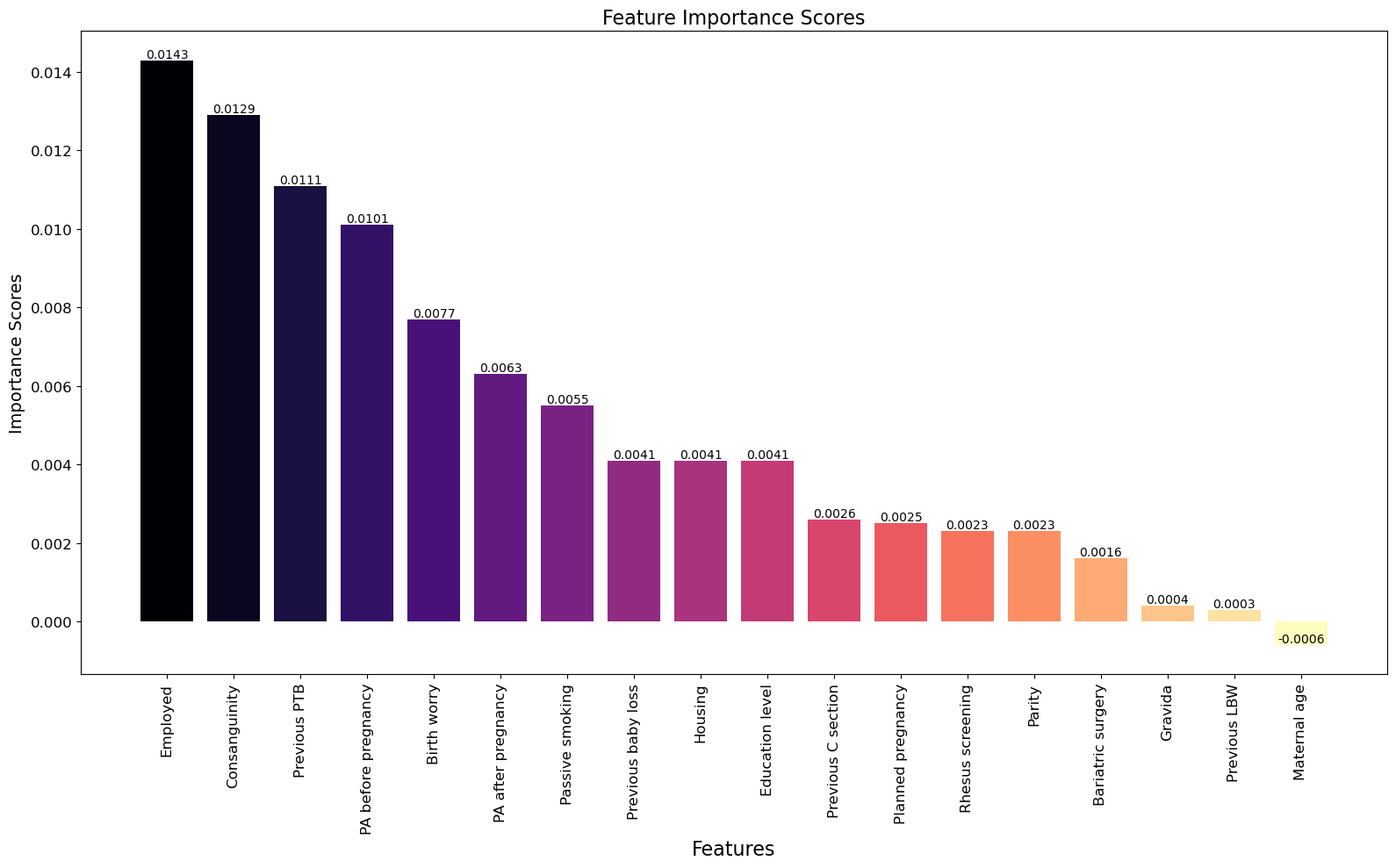


Figure 5. Feature contribution calculation by Anomaly perturbation (weight=2895)

Table 5. Top 5 important features by Anomaly perturbation (weight=2895)

| **Rank** | **Feature** | **Importance Score** |
| --- | --- | --- |
| 1 | Employed | 0.0143 |
| 2 | Consanguinity | 0.0129 |
| 3 | Previous PTB | 0.0111 |
| 4 | PA before pregnancy | 0.0101 |
| 5 | Birth worry | 0.0077 |


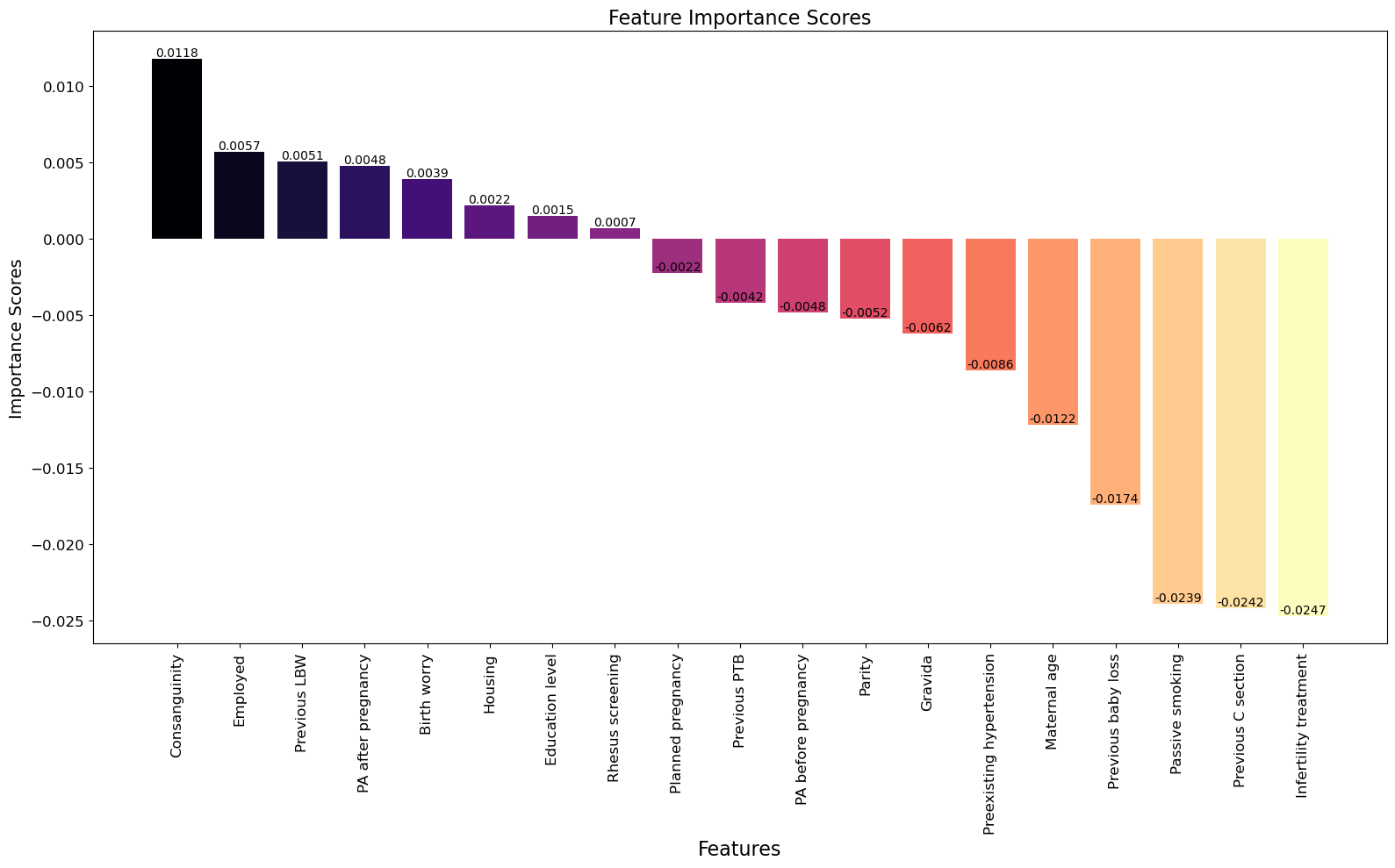


Figure 6. Feature contribution calculation by Anomaly perturbation (weight=2105)

Table 6. Top 5 important features by Anomaly perturbation (weight=2105)

| **Rank** | **Feature** | **Importance Score** |
| --- | --- | --- |
| 1 | Consanguinity | 0.0118 |
| 2 | Employed | 0.0057 |
| 3 | Previous LBW | 0.0051 |
| 4 | PA during pregnancy | 0.0048 |
| 5 | Birth worry | 0.0039 |


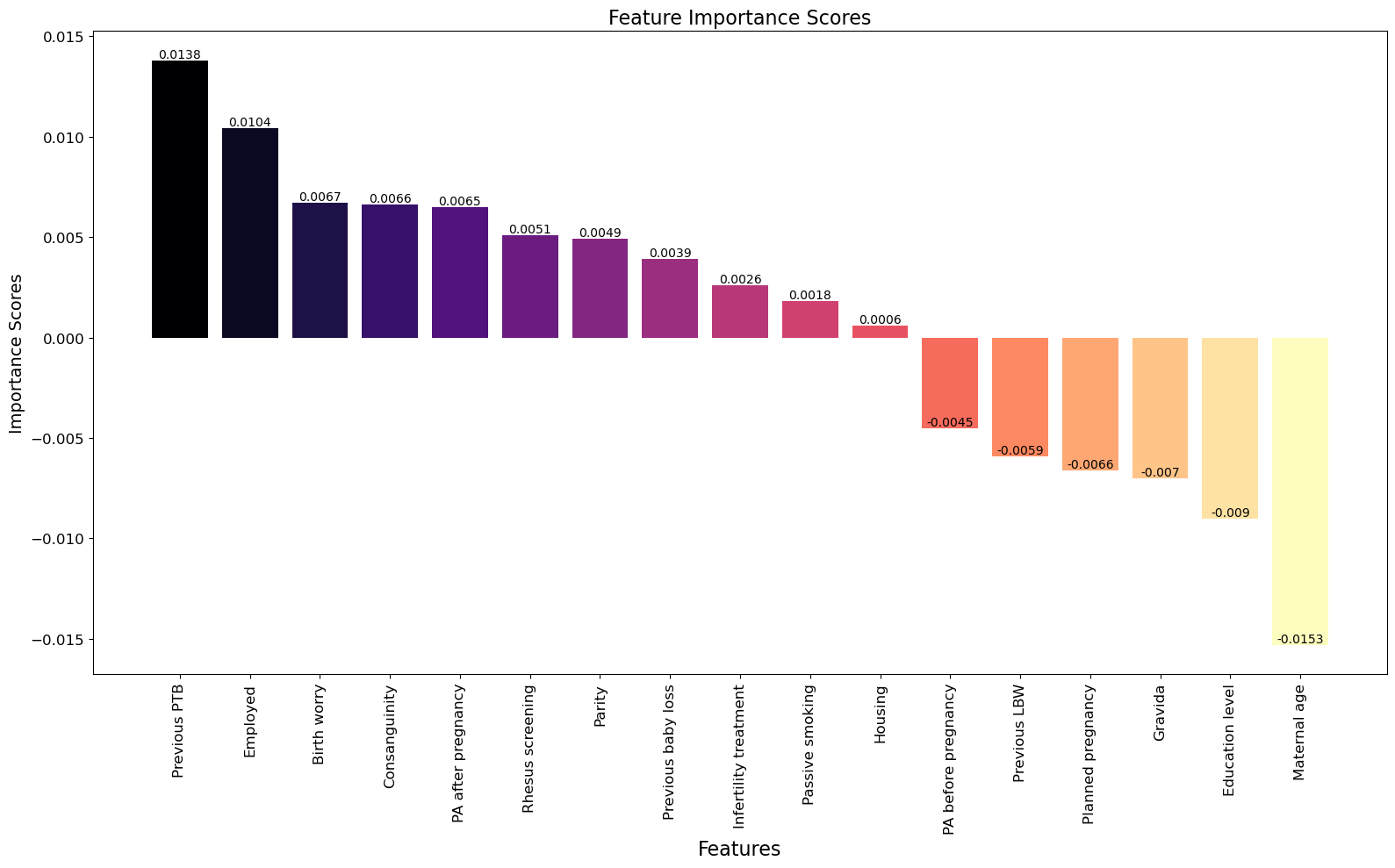


Figure 7. Feature contribution calculation by Anomaly perturbation (weight=1060)

Table 7. Top 5 important features by Anomaly perturbation (weight=1060)

| **Rank** | **Feature** | **Importance Score** |
| --- | --- | --- |
| 1 | Previous PTB | 0.0138 |
| 2 | Employed | 0.0104 |
| 3 | Birth worry | 0.0067 |
| 4 | Consanguinity | 0.0066 |
| 5 | PA during pregnancy | 0.0065 |


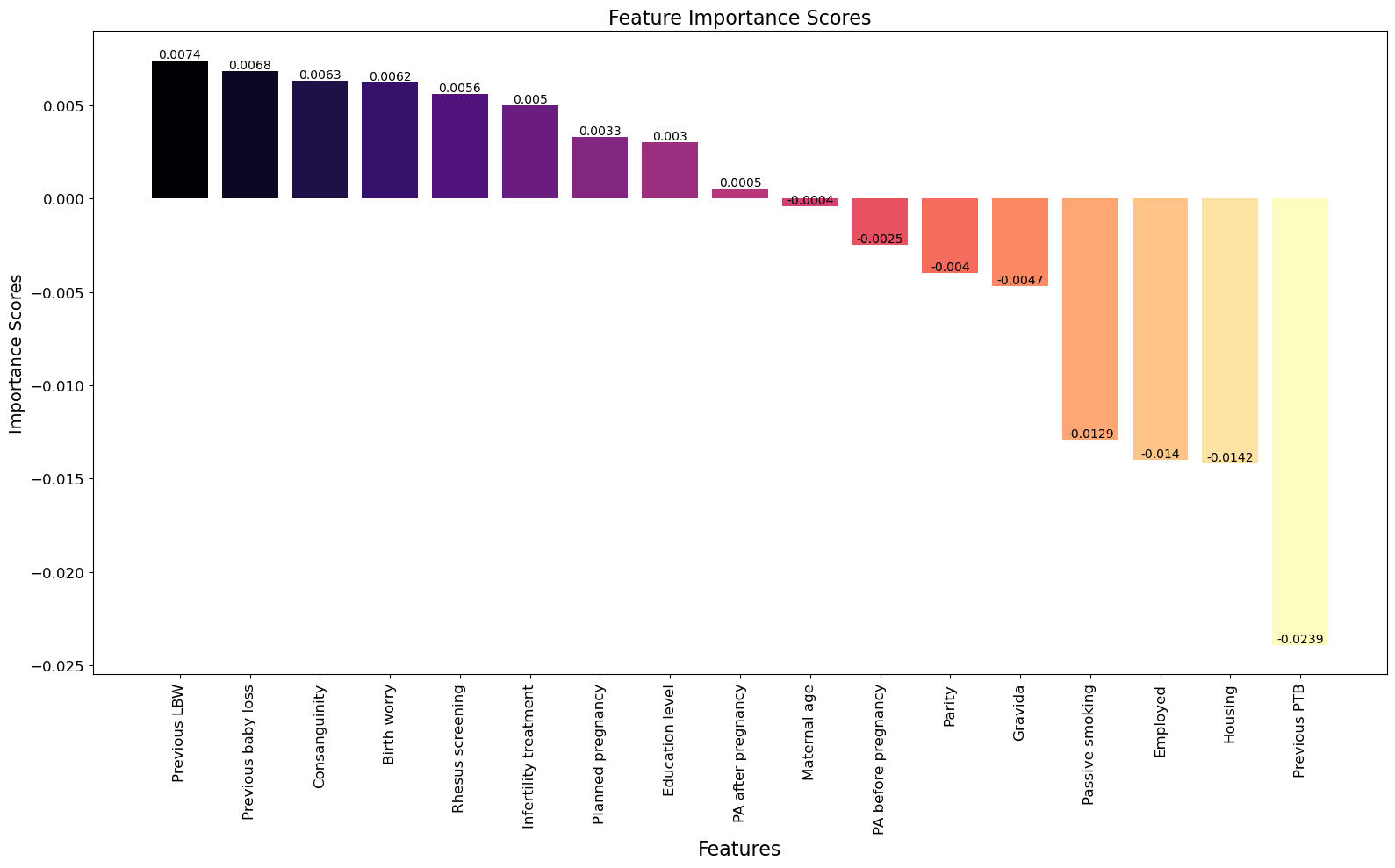


Figure 8. Feature contribution calculation by Anomaly perturbation (weight=465)

Table 8. Top 5 important features by Anomaly perturbation (weight=465)

| **Rank** | **Feature** | **Importance Score** |
| --- | --- | --- |
| 1 | Previous LBW | 0.0074 |
| 2 | Previous PTB | 0.0068 |
| 3 | Consanguinity | 0.0063 |
| 4 | Birth worry | 0.0062 |
| 5 | Rhesus screening | 0.0056 |

**Very Low Birth Weight**


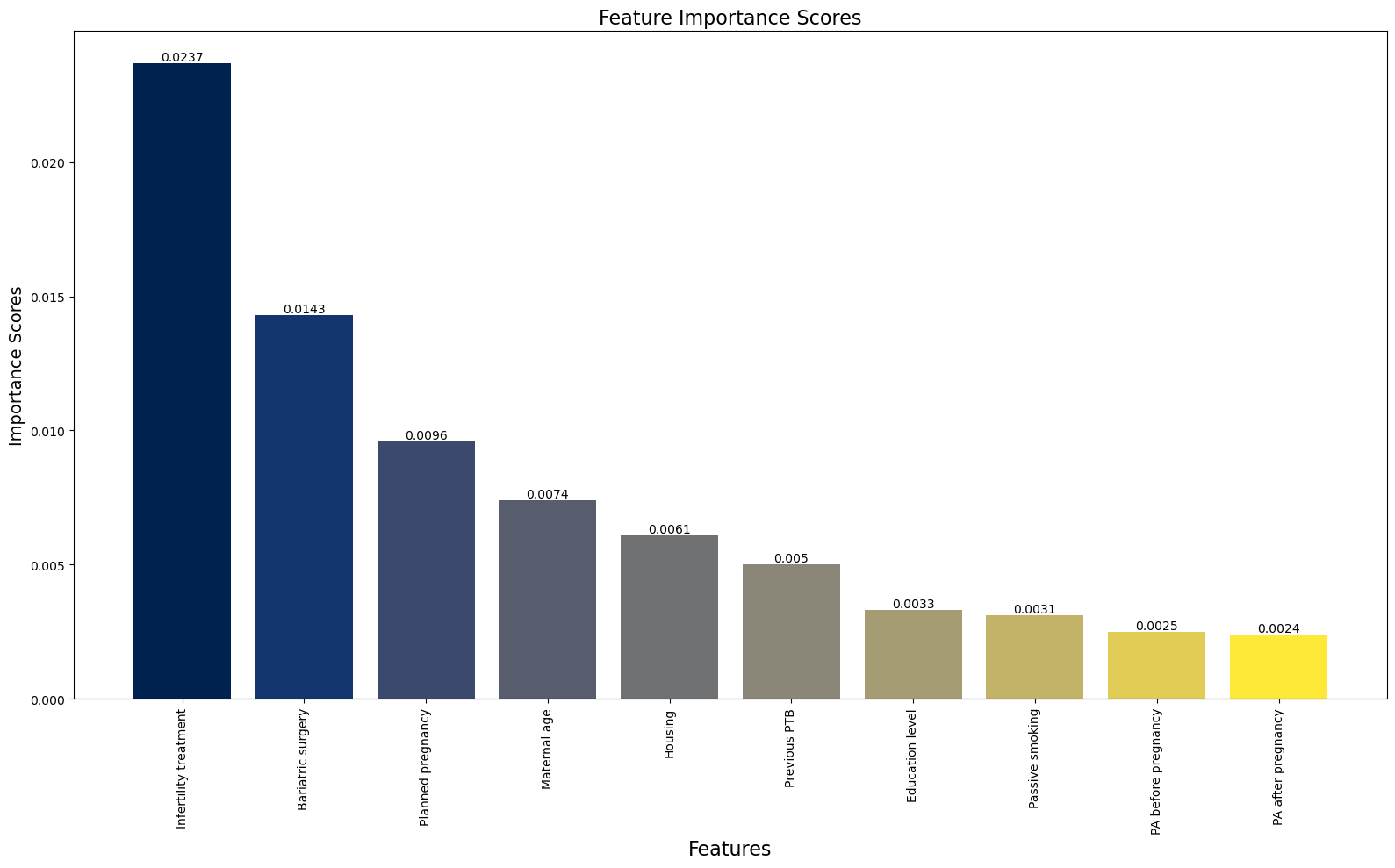


Figure 9. Feature contribution calculation by Local-DIFFI (weight=2895)

Table 9. Top 5 important features by Local-DIFFI (weight=2895)

| **Rank** | **Feature** | **Importance Score** |
| --- | --- | --- |
| 1 | Infertility treatment | 0.0237 |
| 2 | Bariatric surgery | 0.0143 |
| 3 | Planned pregnancy | 0.0096 |
| 4 | Maternal age | 0.0074 |
| 5 | Housing | 0.0061 |


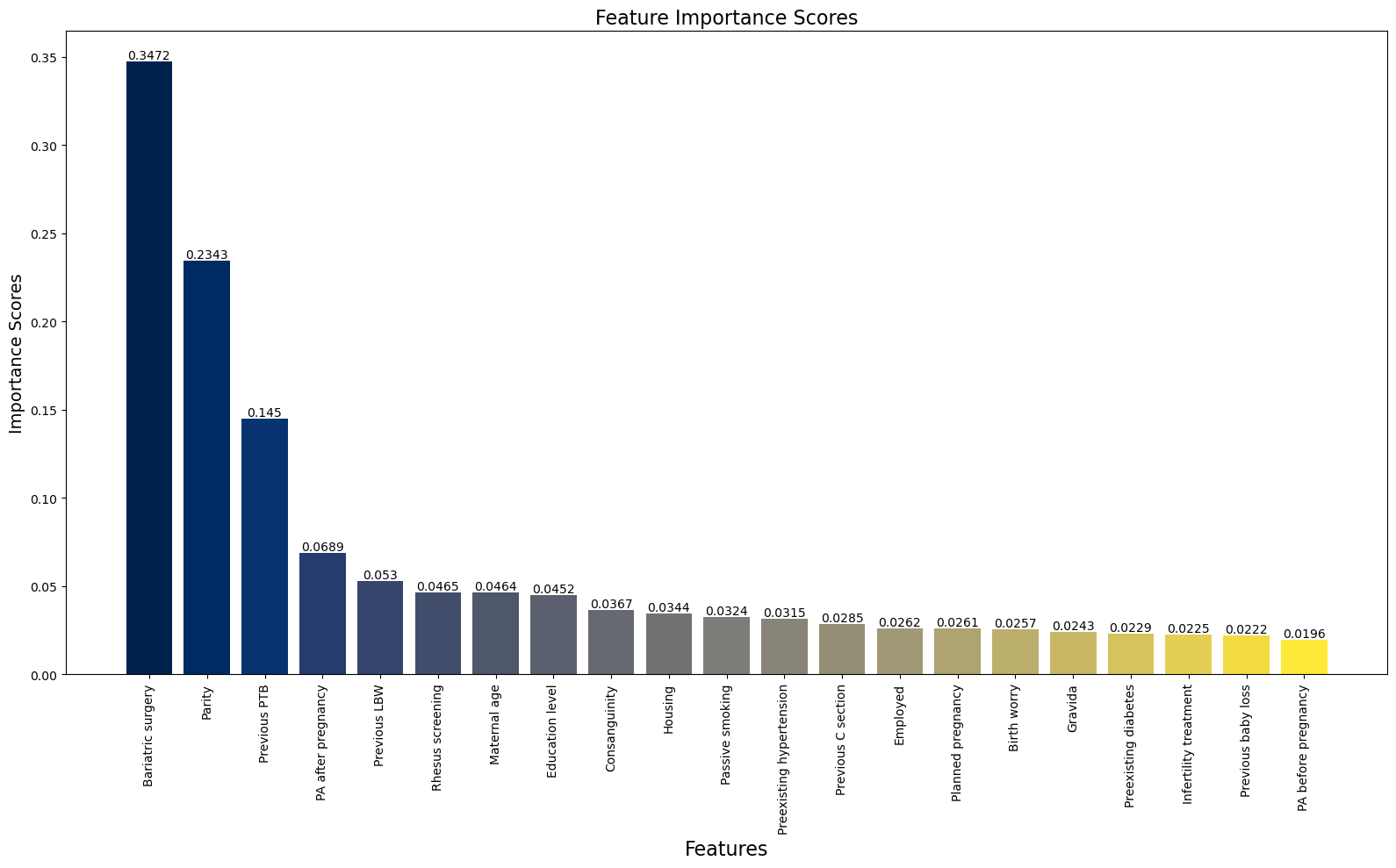


Figure 10. Feature contribution calculation by Local-DIFFI (weight=2105)

Table 10. Top 5 important features by Local-DIFFI (weight=2105)

| **Rank** | **Feature** | **Importance Score** |
| --- | --- | --- |
| 1 | Bariatric surgery | 0.3472 |
| 2 | Parity | 0.2343 |
| 3 | Previous PTB | 0.145 |
| 4 | PA during pregnancy | 0.0689 |
| 5 | Previous LBW | 0.053 |


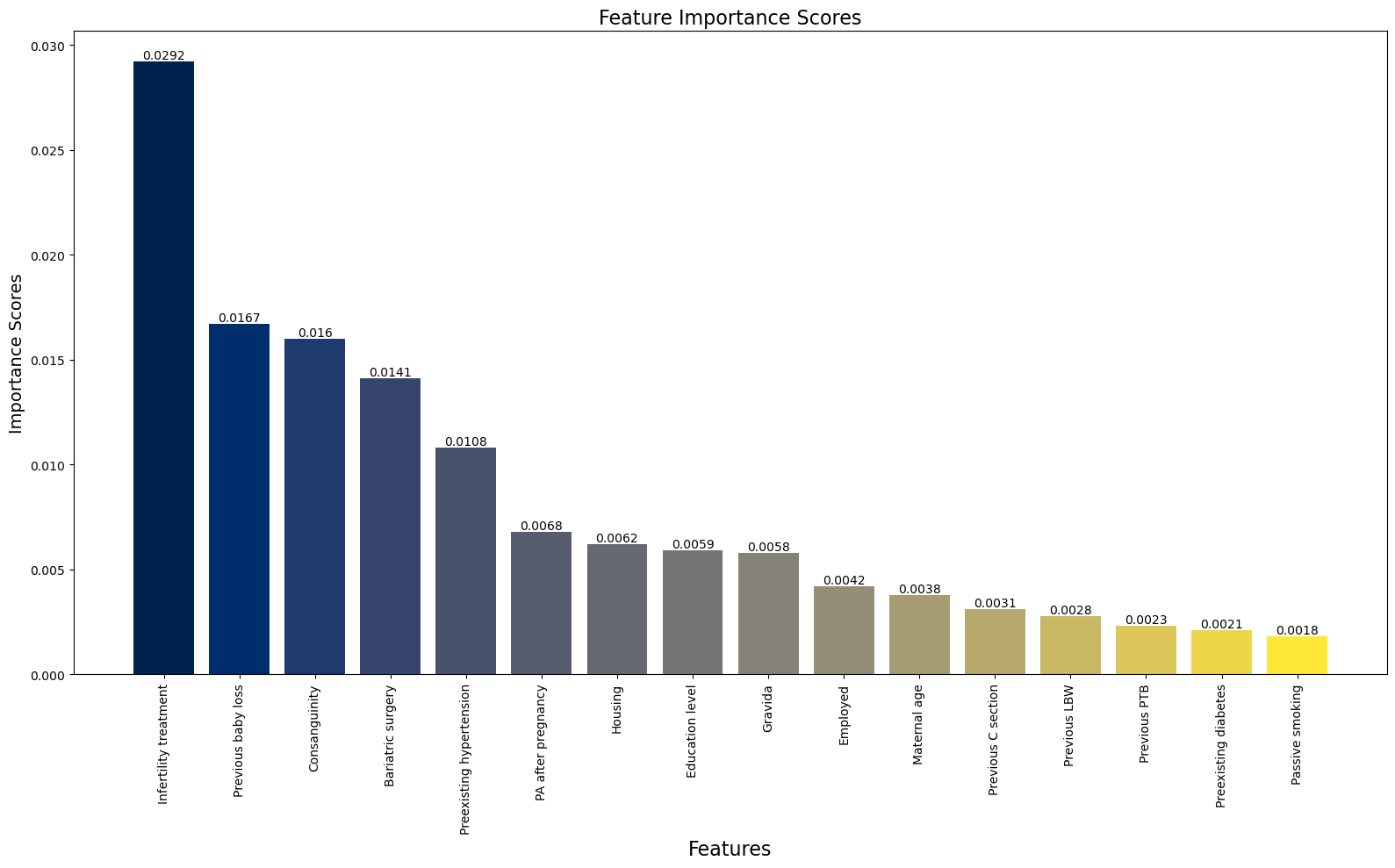


Figure 11. Feature contribution calculation by Local-DIFFI (weight=1060)

Table 11. Top 5 important features by Local-DIFFI (weight=1060)

| **Rank** | **Feature** | **Importance Score** |
| --- | --- | --- |
| 1 | Infertility treatment | 0.0292 |
| 2 | Previous baby Loss | 0.0167 |
| 3 | Consanguinity | 0.016 |
| 4 | Bariatric surgery | 0.0141 |
| 5 | Preexisting hypertension | 0.0108 |


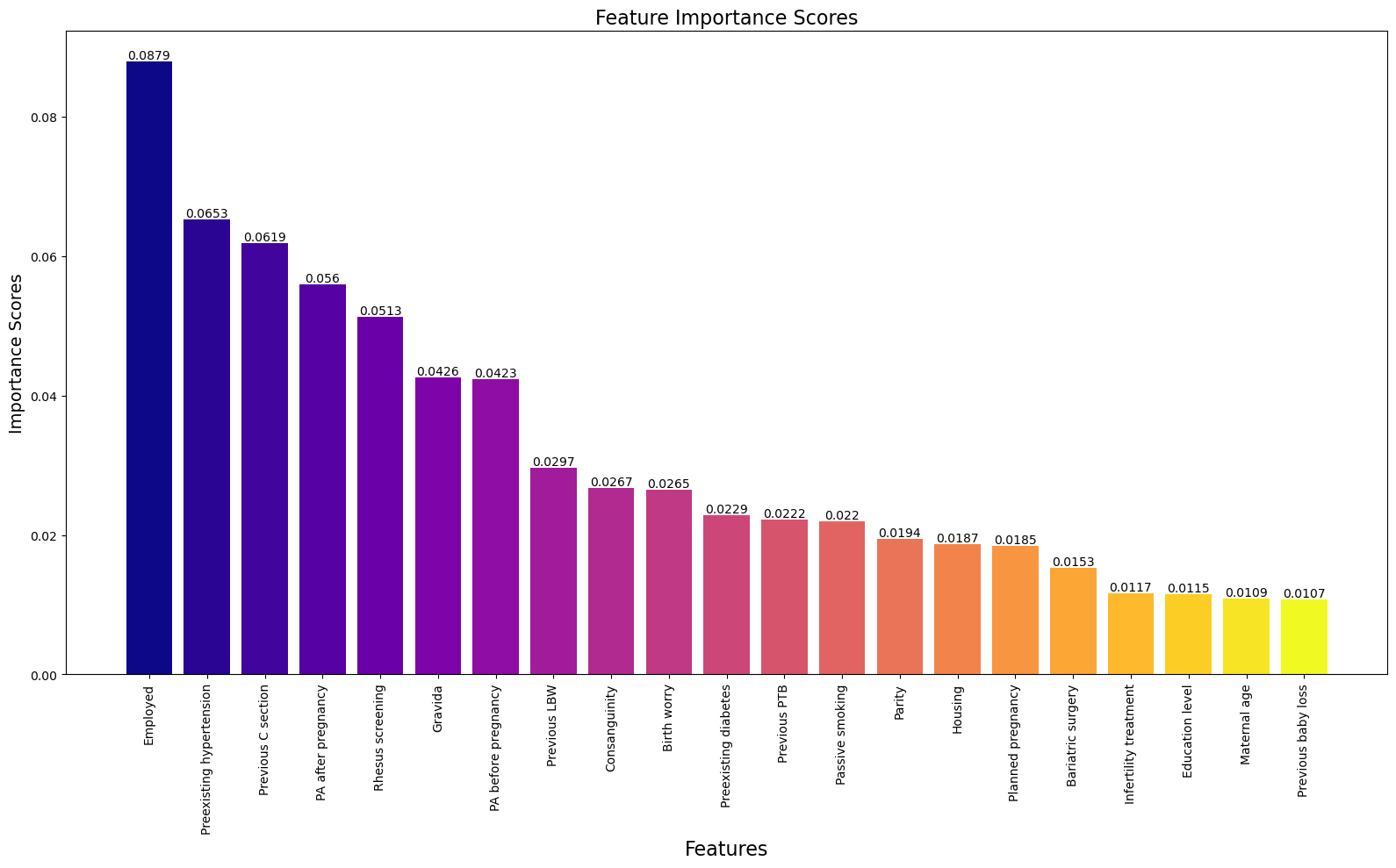


Figure 12. Feature contribution calculation by Local-DIFFI (weight=465)

Table 12. Top 5 important features by Local-DIFFI (weight=465)

| **Rank** | **Feature** | **Importance Score** |
| --- | --- | --- |
| 1 | Employed | 0.0879 |
| 2 | Preexisting hypertension | 0.0653 |
| 3 | Previous C section | 0.0619 |
| 4 | PA during pregnancy | 0.056 |
| 5 | Rhesus screeing | 0.0513 |


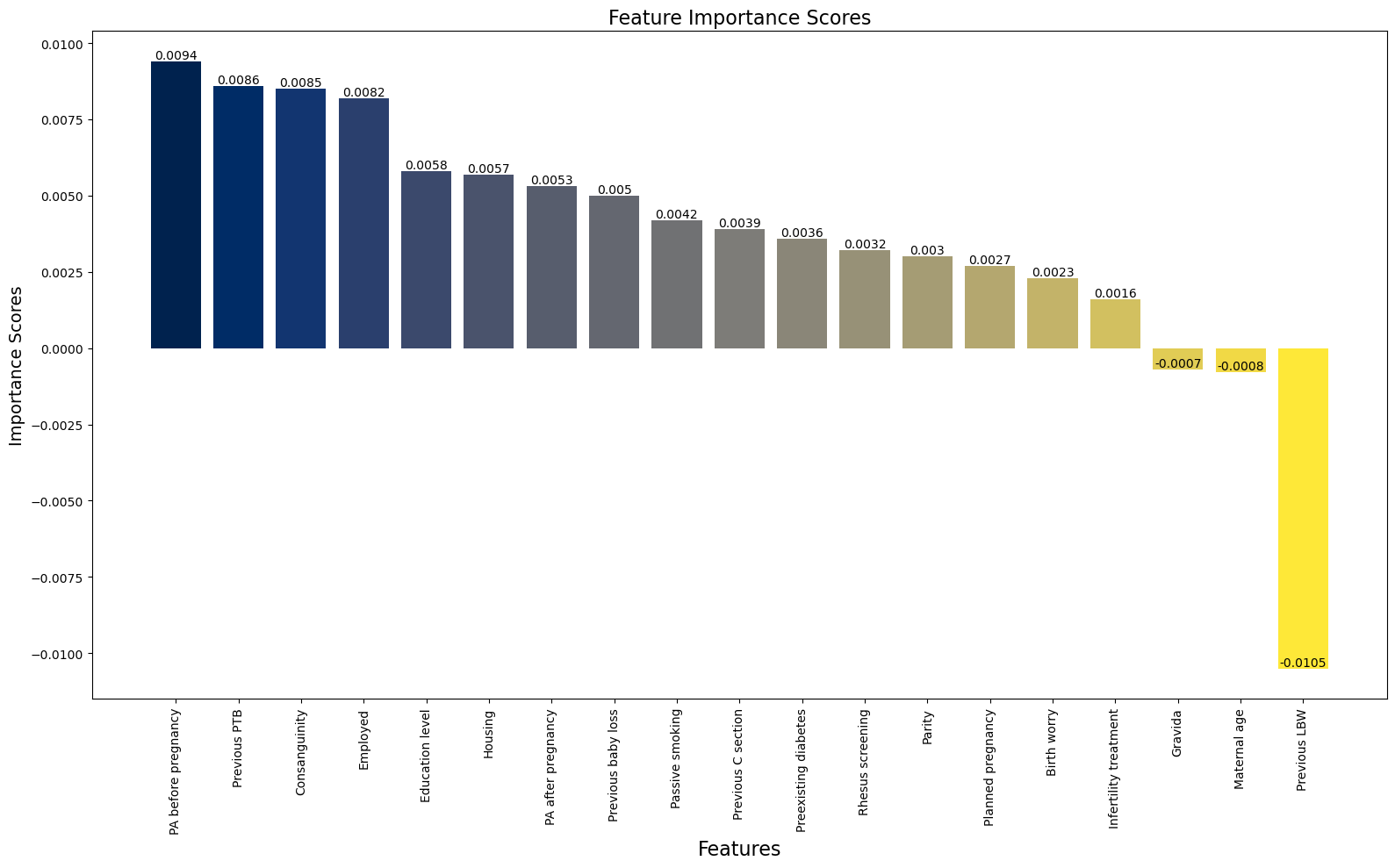


Figure 13. Feature contribution calculation by Anomaly perturbation (weight=2895)

Table 13. Top 5 important features by Anomaly perturbation (weight=2895)

| **Rank** | **Feature** | **Importance Score** |
| --- | --- | --- |
| 1 | PA before pregnancy | 0.0094 |
| 2 | Previous PTB | 0.0086 |
| 3 | Consanguinity | 0.0085 |
| 4 | Employed | 0.0082 |
| 5 | Education level | 0.0058 |


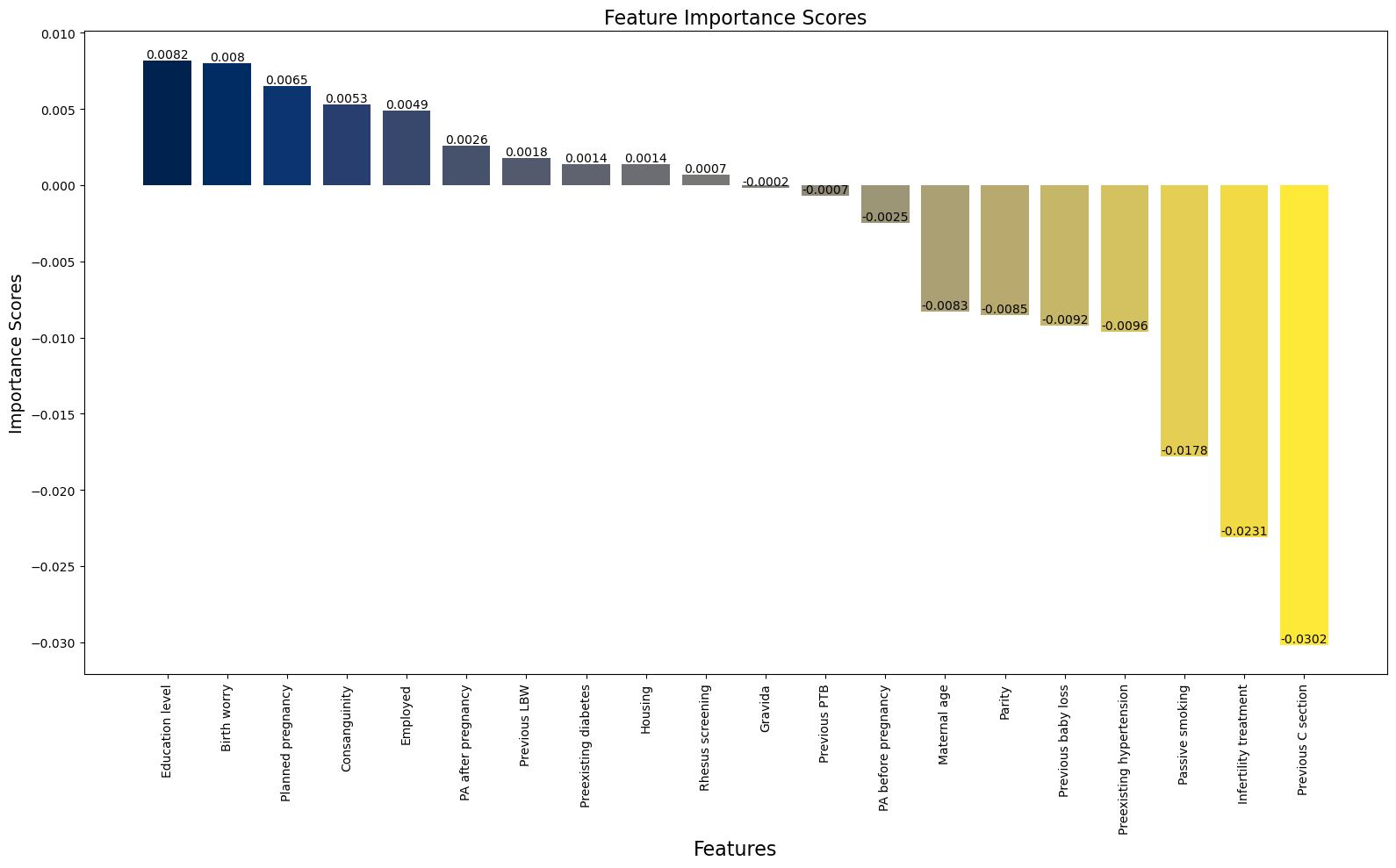


Figure 14. Feature contribution calculation by Anomaly perturbation (weight=2105)

Table 14. Top 5 important features by Anomaly perturbation (weight=2105)

| **Rank** | **Feature** | **Importance Score** |
| --- | --- | --- |
| 1 | Education level | 0.0082 |
| 2 | Birth worry | 0.008 |
| 3 | Planned pregnancy | 0.0065 |
| 4 | Consanguinity | 0.0053 |
| 5 | Employed | 0.0049 |


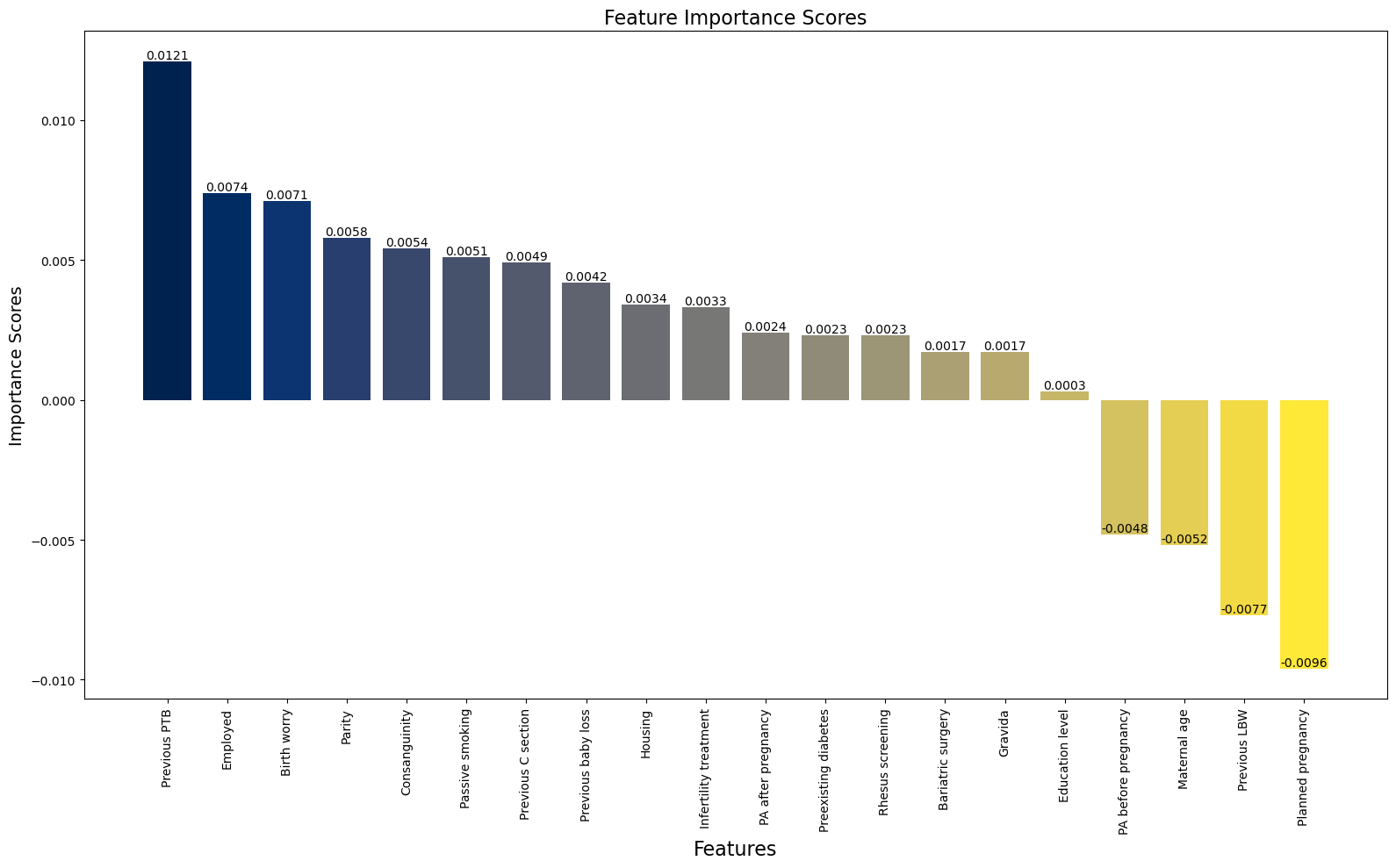


Figure 15. Feature contribution calculation by Anomaly perturbation (weight=1060)

Table 15. Top 5 important features by Anomaly perturbation (weight=1060)

| **Rank** | **Feature** | **Importance Score** |
| --- | --- | --- |
| 1 | Previous PTB | 0.0121 |
| 2 | Employed | 0.0074 |
| 3 | Birth worry | 0.0071 |
| 4 | Parity | 0.0058 |
| 5 | Consanguinity | 0.0054 |


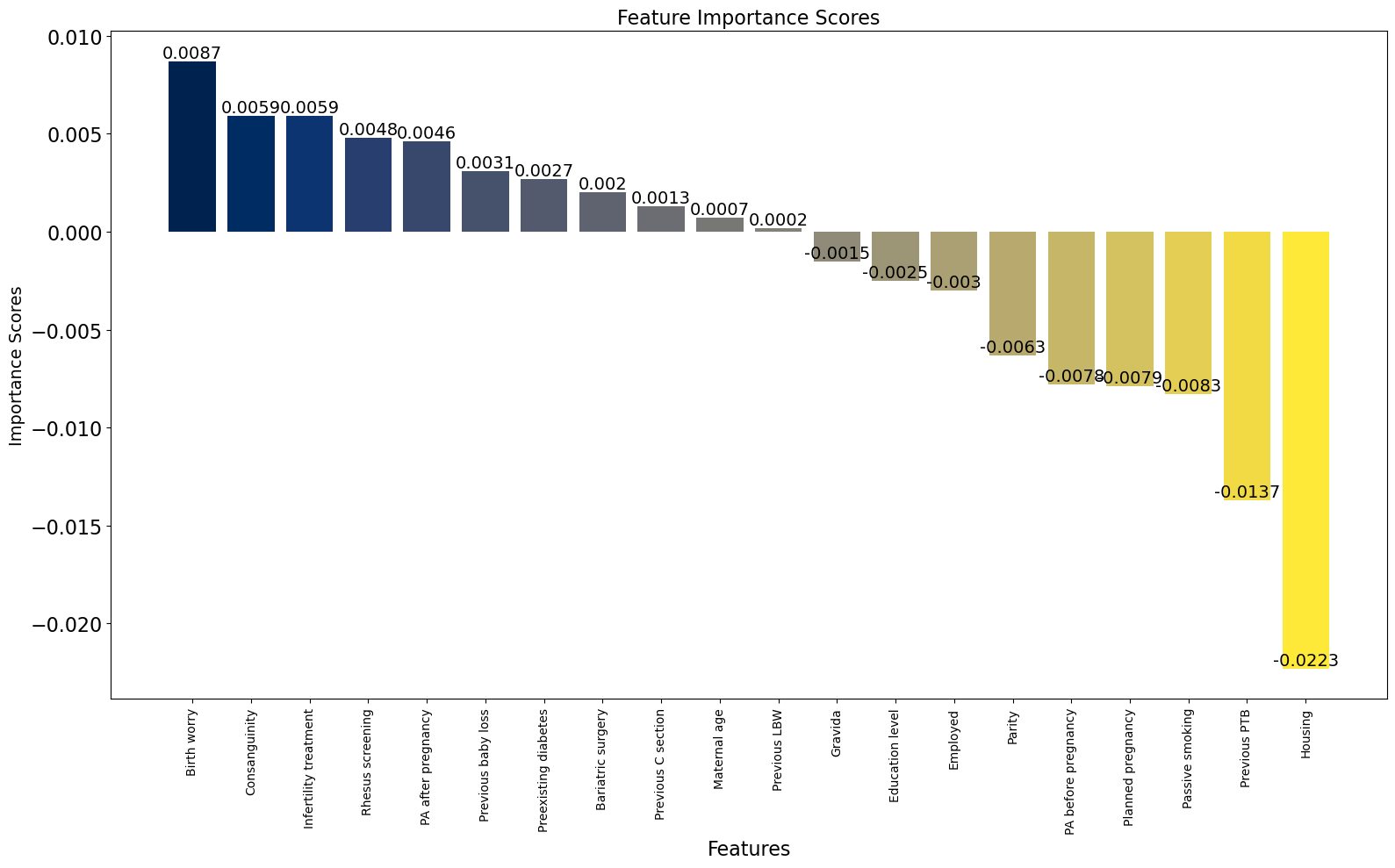


Figure 16. Feature contribution calculation by Anomaly perturbation (weight=465)

Table 16. Top 5 important features by Anomaly perturbation (weight=465)

| **Rank** | **Feature** | **Importance Score** |
| --- | --- | --- |
| 1 | Birth worry | 0.0087 |
| 2 | Consanguinity | 0.0059 |
| 3 | Infertility treatment | 0.0059 |
| 4 | Rhesus screening | 0.0048 |
| 5 | PA during pregnancy | 0.0046 |

**Extreme Low Birth Weight**


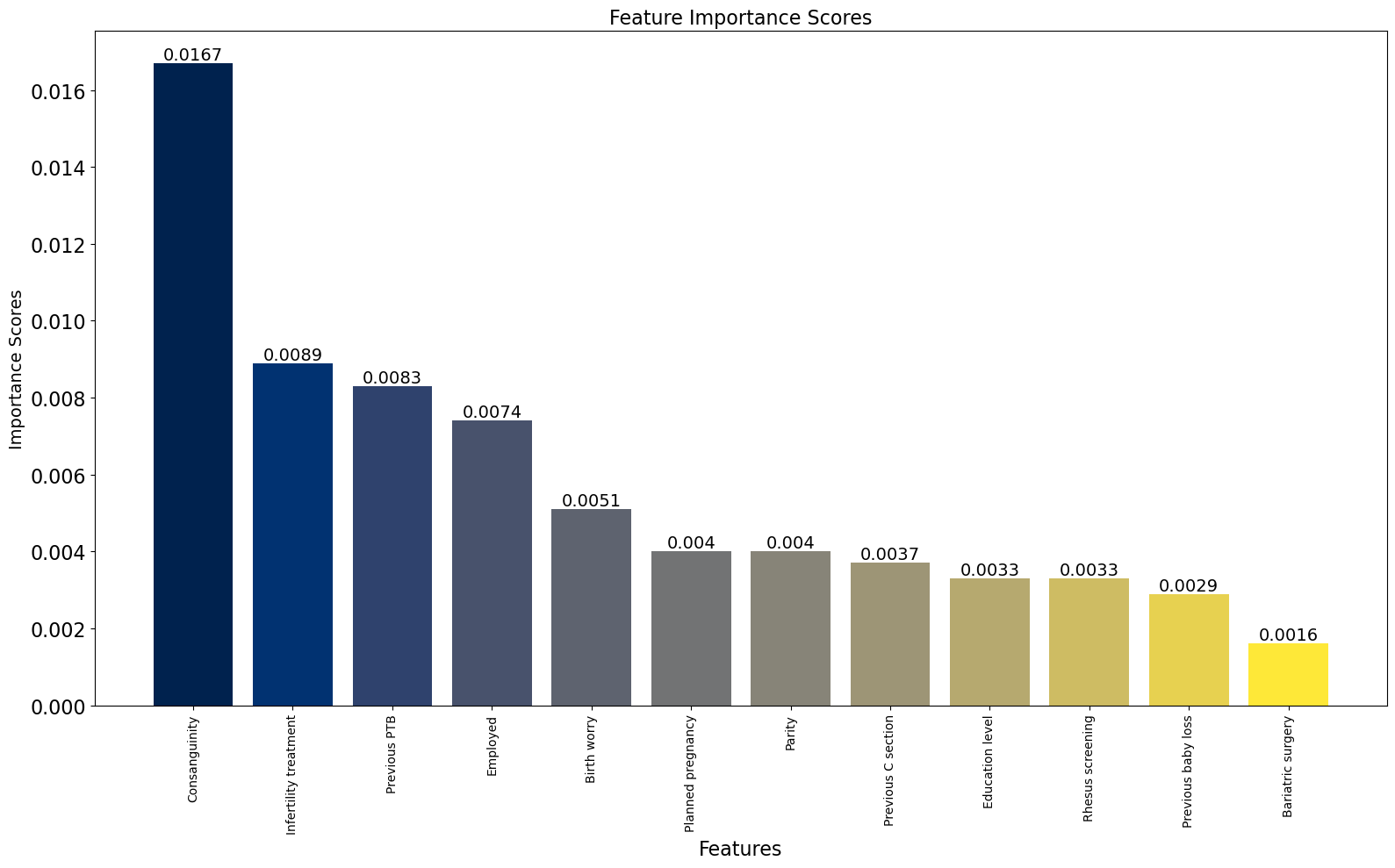


Figure 17. Feature contribution calculation by Local-DIFFI (weight=2895)

Table 17. Top 5 important features by Local-DIFFI (weight=2895)

| **Rank** | **Feature** | **Importance Score** |
| --- | --- | --- |
| 1 | Consanguinity | 0.0167 |
| 2 | Infertility treatment | 0.0089 |
| 3 | Previous PTB | 0.0083 |
| 4 | Employed | 0.0074 |
| 5 | Birth worry | 0.0051 |


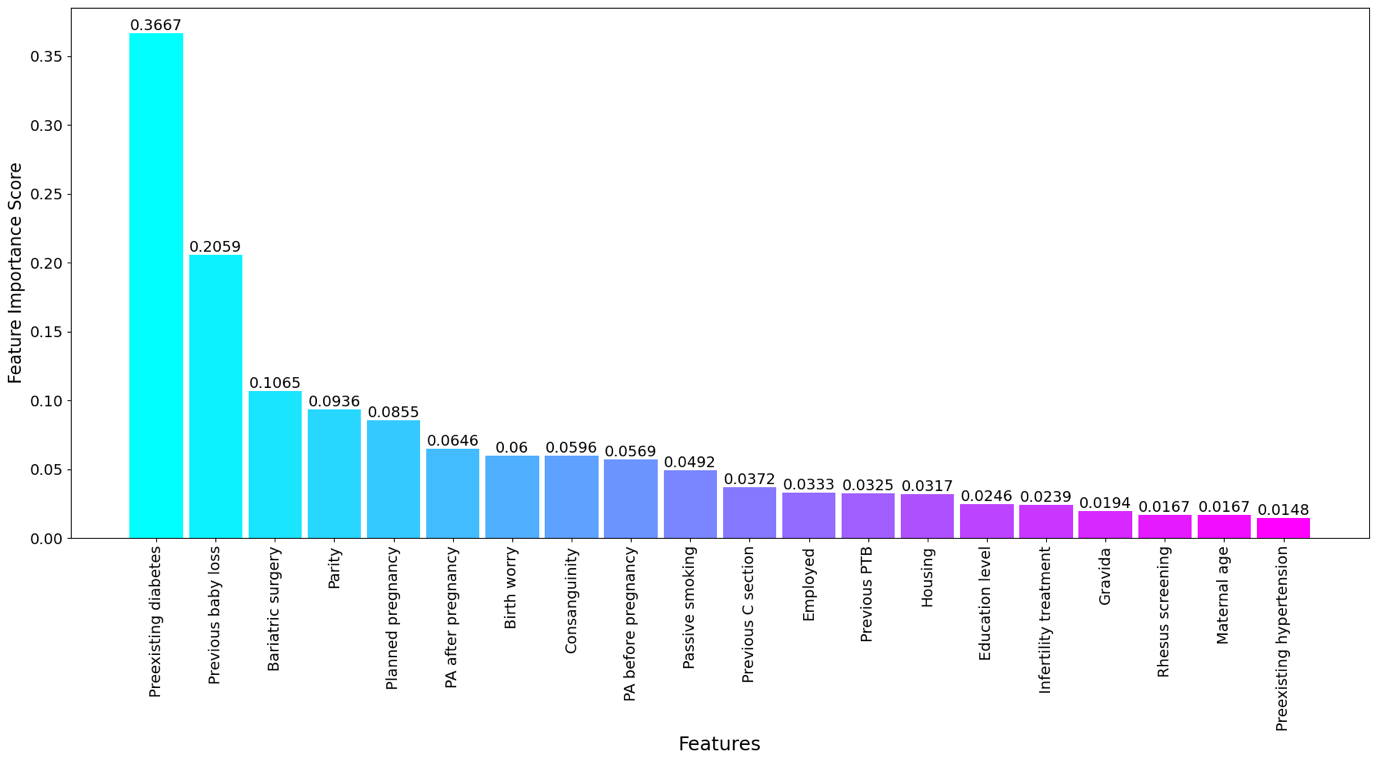


Figure 18. Feature contribution calculation by Local-DIFFI (weight=2105)

Table 18. Top 5 important features by Local-DIFFI (weight=2105)

| **Rank** | **Feature** | **Importance Score** |
| --- | --- | --- |
| 1 | Preexisting diabetes | 0.3667 |
| 2 | Previous baby Loss | 0.2059 |
| 3 | Bariatric surgery | 0.1065 |
| 4 | Parity | 0.0936 |
| 5 | Planned pregnancy | 0.0855 |


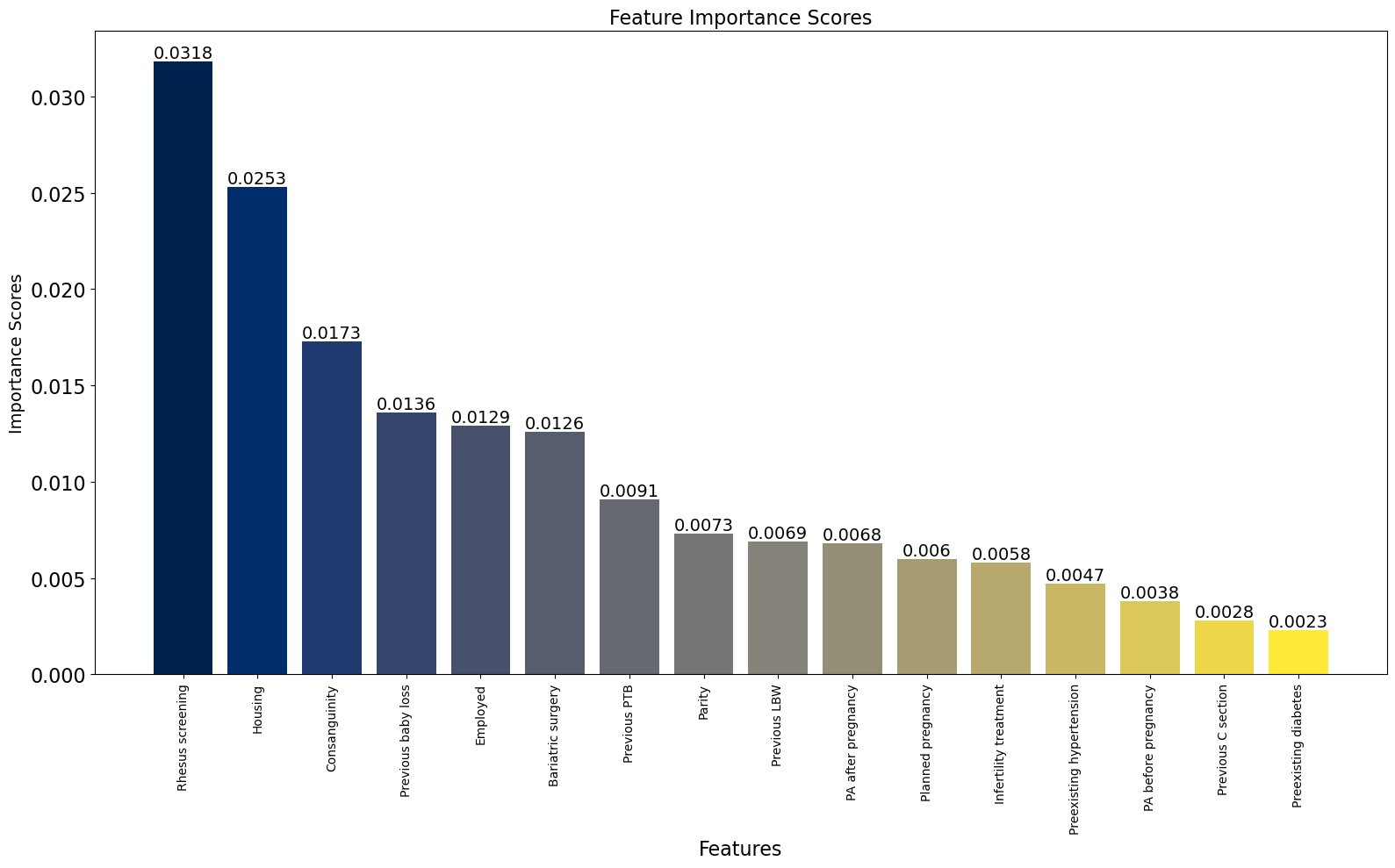


Figure 19. Feature contribution calculation by Local-DIFFI (weight=1065)

Table 19. Top 5 important features by Local-DIFFI (weight=1065)

| **Rank** | **Feature** | **Importance Score** |
| --- | --- | --- |
| 1 | Rhesus screening | 0.0318 |
| 2 | Housing | 0.0253 |
| 3 | Consanguinity | 0.0173 |
| 4 | Previous baby Loss | 0.0136 |
| 5 | Employed | 0.0129 |


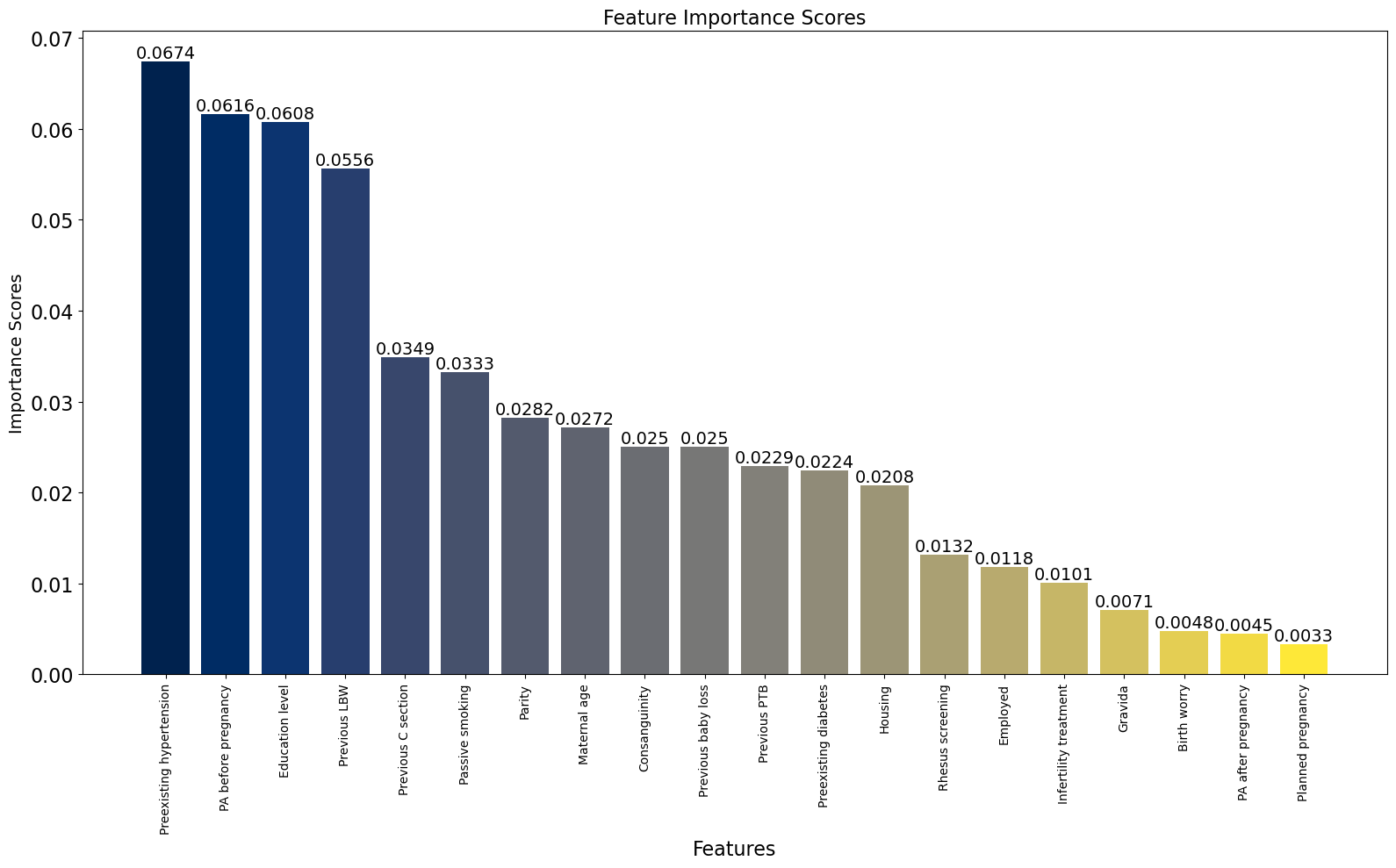


Figure 20. Feature contribution calculation by Local-DIFFI (weight=465)

Table 20. Top 5 important features by Local-DIFFI (weight=465)

| **Rank** | **Feature** | **Importance Score** |
| --- | --- | --- |
| 1 | Preexisting hypertension | 0.0674 |
| 2 | PA before pregnancy | 0.0616 |
| 3 | Education level | 0.0608 |
| 4 | Previous LBW | 0.0556 |
| 5 | Previous C section | 0.0349 |


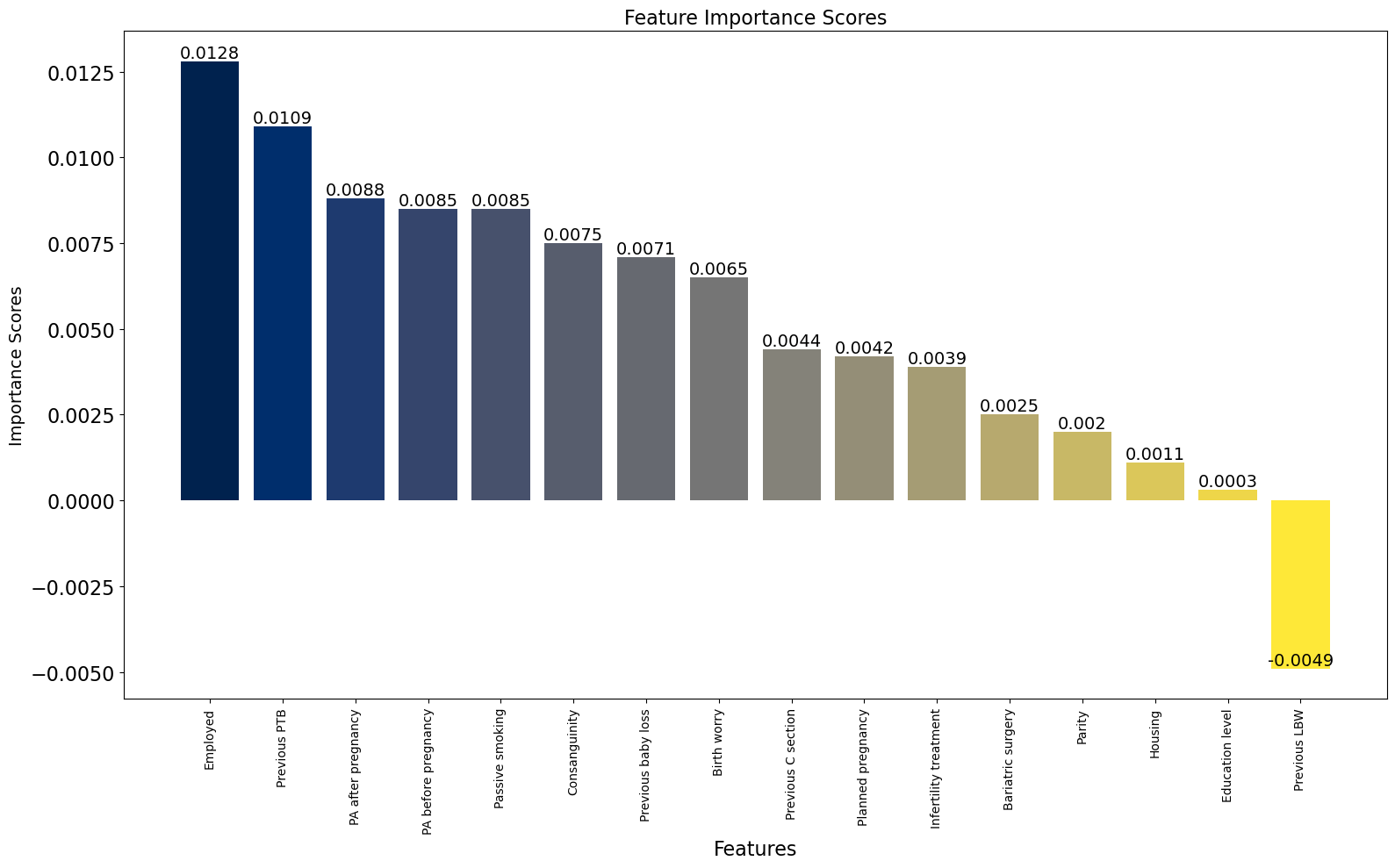


Figure 21. Feature contribution calculation by Anomaly perturbation (weight=2895)

Table 21. Top 5 important features by Anomaly perturbation (weight=2895)

| **Rank** | **Feature** | **Importance Score** |
| --- | --- | --- |
| 1 | Employed | 0.0128 |
| 2 | Previous PTB | 0.0109 |
| 3 | PA during pregnancy | 0.0088 |
| 4 | PA before pregnancy | 0.0085 |
| 5 | Passive smoking | 0.0085 |


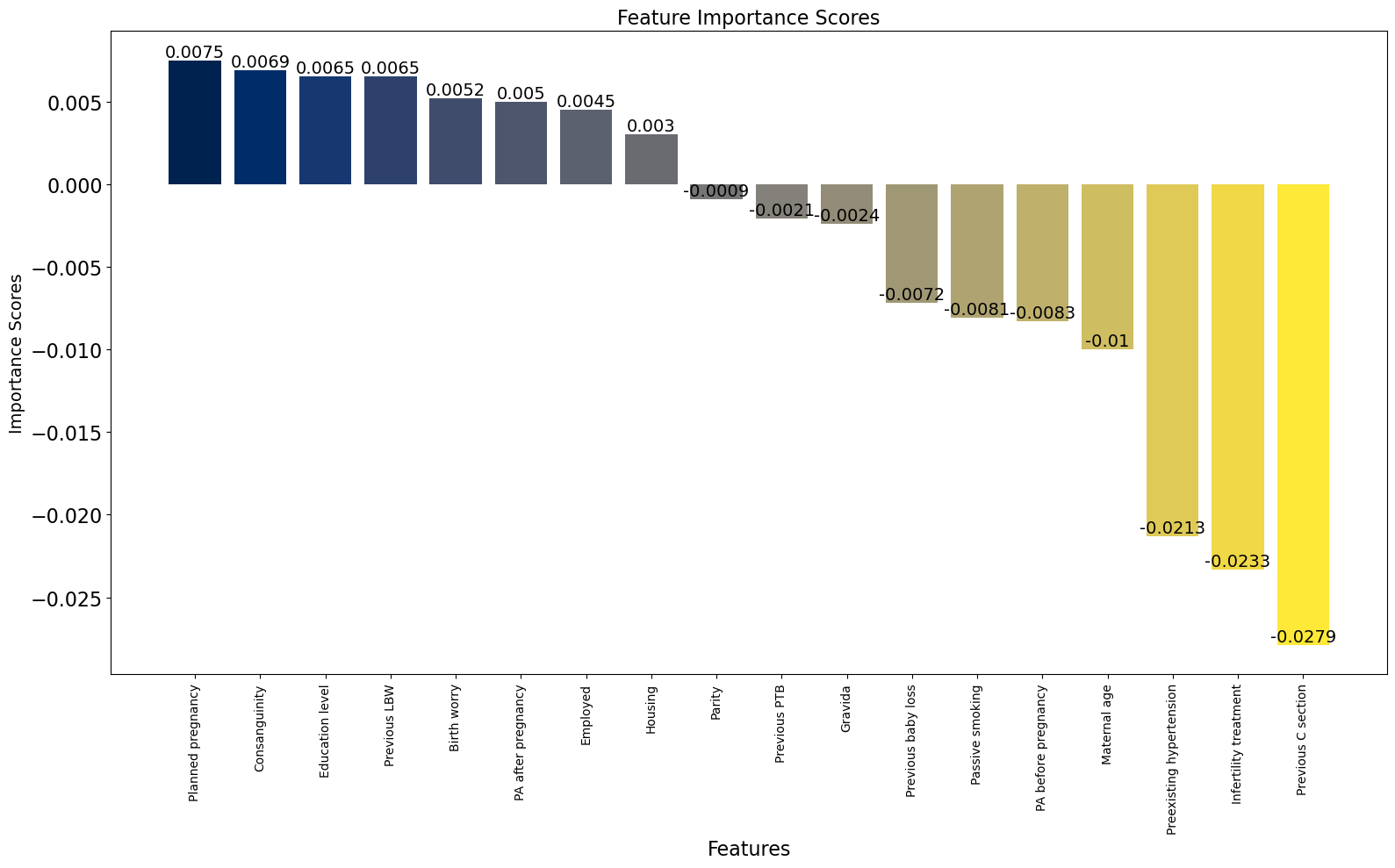


Figure 22. Feature contribution calculation by Anomaly perturbation (weight=2105)

Table 22. Top 5 important features by Anomaly perturbation (weight=2105)

| **Rank** | **Feature** | **Importance Score** |
| --- | --- | --- |
| 1 | Planned pregnancy | 0.0075 |
| 2 | Consanguinity | 0.0069 |
| 3 | Education level | 0.0065 |
| 4 | Previous LBW | 0.0065 |
| 5 | Birth worry | 0.0052 |


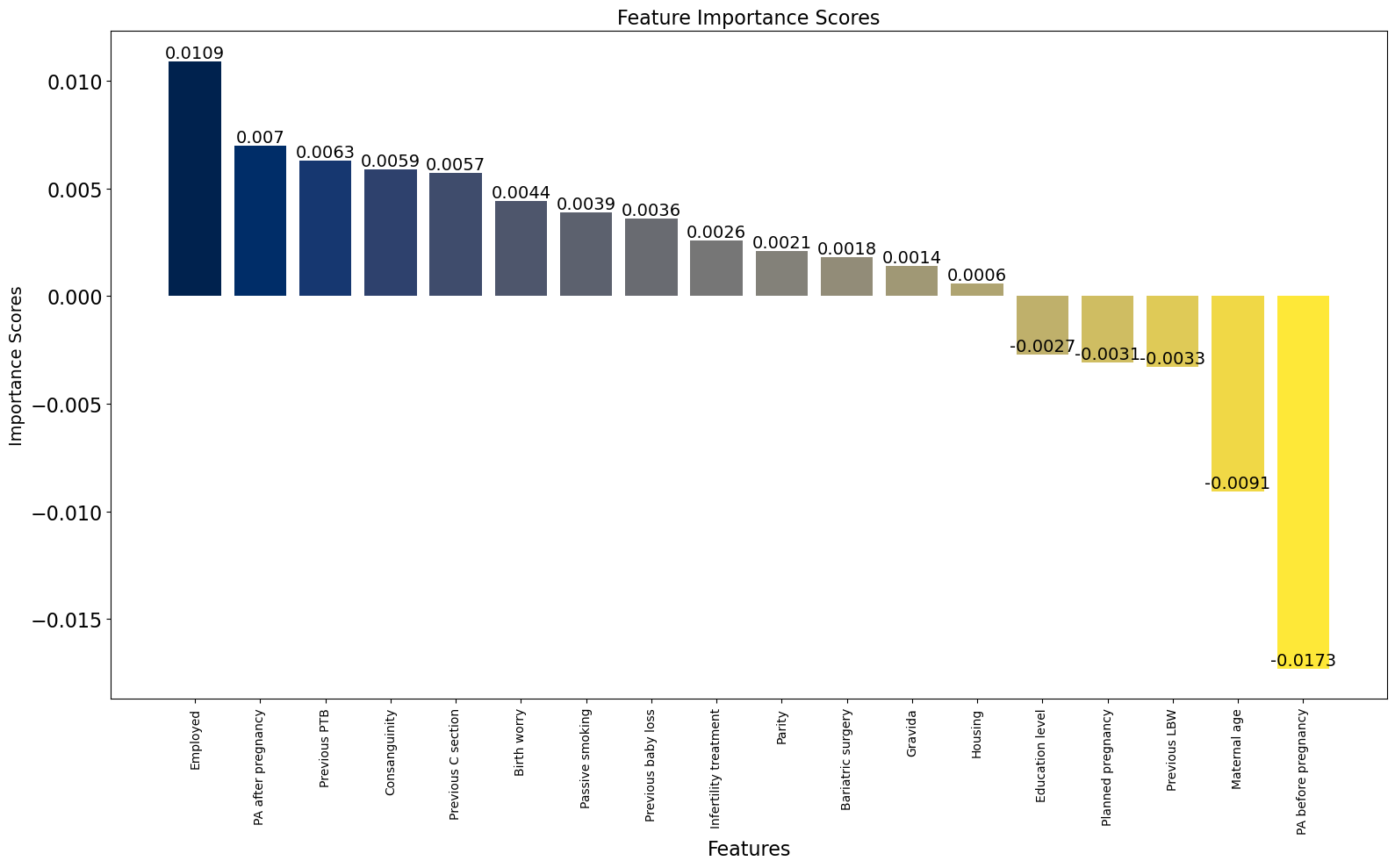


Figure 23. Feature contribution calculation by Anomaly perturbation (weight=1060)

Table 23. Top 5 important features by Anomaly perturbation (weight=1060)

| **Rank** | **Feature** | **Importance Score** |
| --- | --- | --- |
| 1 | Employed | 0.0109 |
| 2 | PA during pregnancy | 0.007 |
| 3 | Previous PTB | 0.0063 |
| 4 | Consanguinity | 0.0059 |
| 5 | Previous C section | 0.0057 |


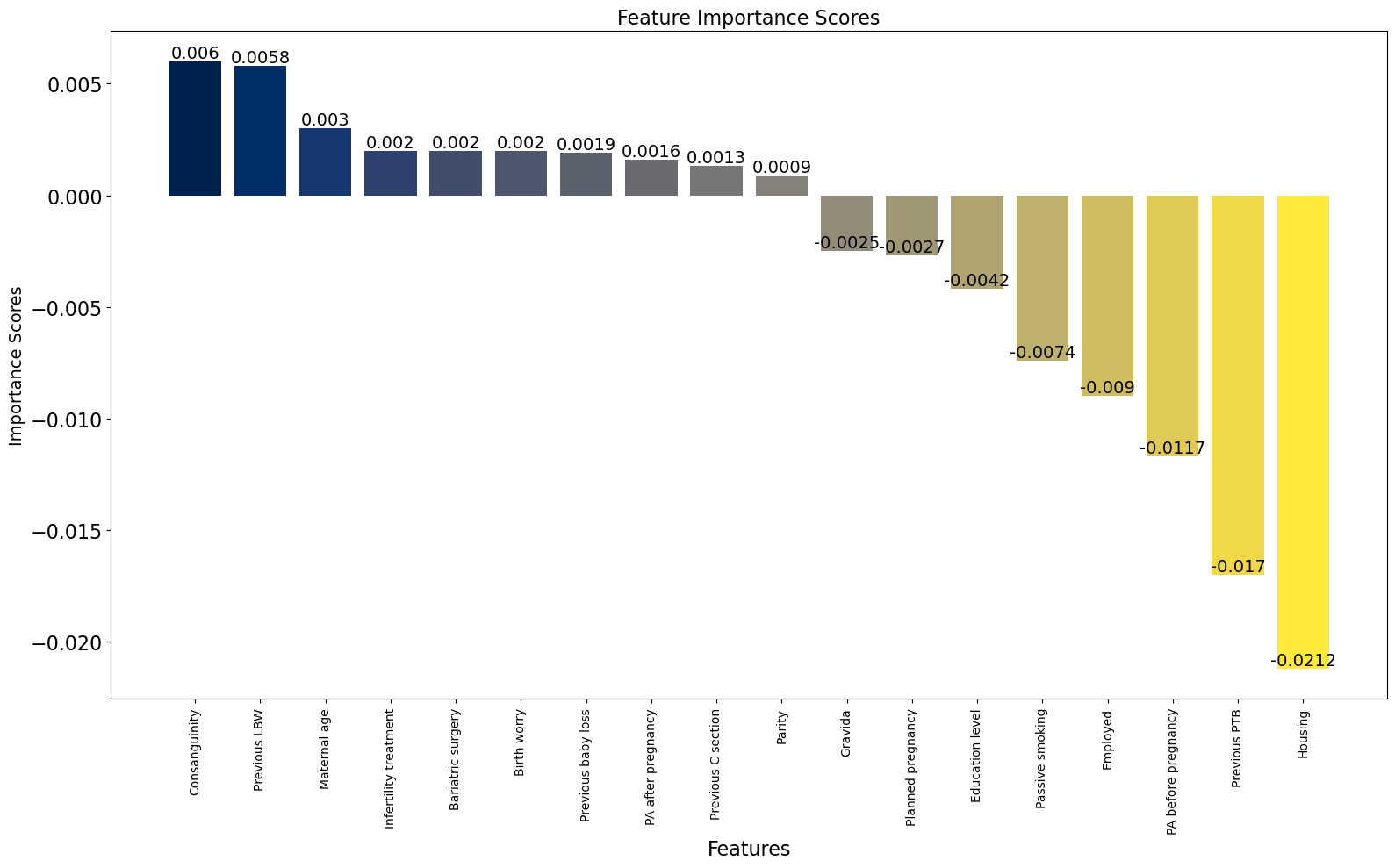


Figure 24. Feature contribution calculation by Anomaly perturbation (weight=465)

Table 24. Top 5 important features by Anomaly perturbation (weight=465)

| **Rank** | **Feature** | **Importance Score** |
| --- | --- | --- |
| 1 | Consanguinity | 0.006 |
| 2 | Previous LBW | 0.0058 |
| 3 | Maternal age | 0.003 |
| 4 | Infertility treatment | 0.002 |
| 5 | Bariatric surgery | 0.002 |

**Average Feature Rank**


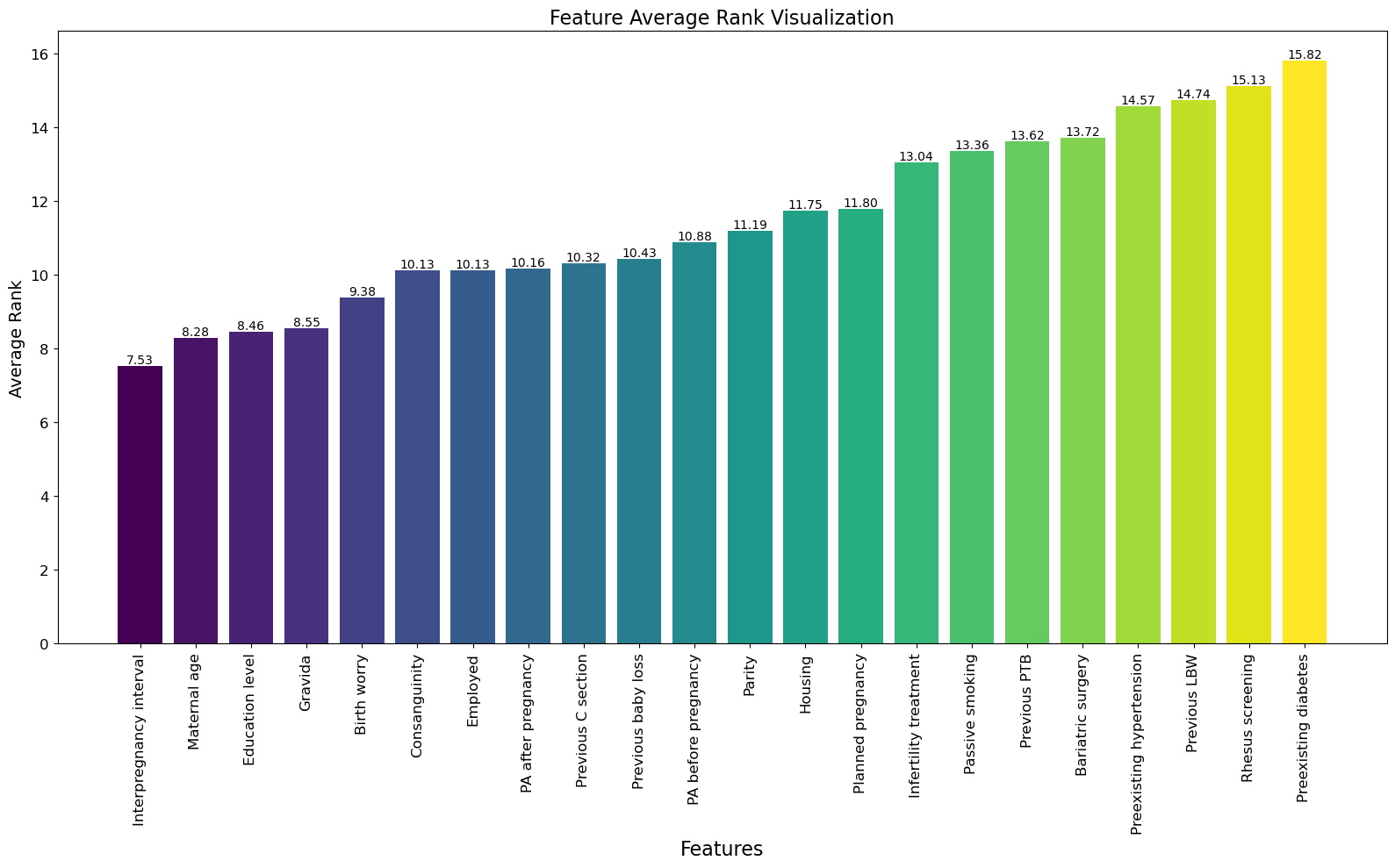


Figure 25. Average rank of all the features across all the outlier points for low birth weight detection using Local-DIFFI

Table 25. Average rank of all the features across all the outlier points for low birth weight detection using Local-DIFFI

| **Rank** | **Feature** | **Importance Score** |
| --- | --- | --- |
| 1 | Preexisting diabetes | 15.82 |
| 2 | Rhesus screening | 15.13 |
| 3 | Previous LBW | 14.74 |
| 4 | Preexisting hypertension | 14.57 |
| 5 | Bariatric surgery | 13.72 |


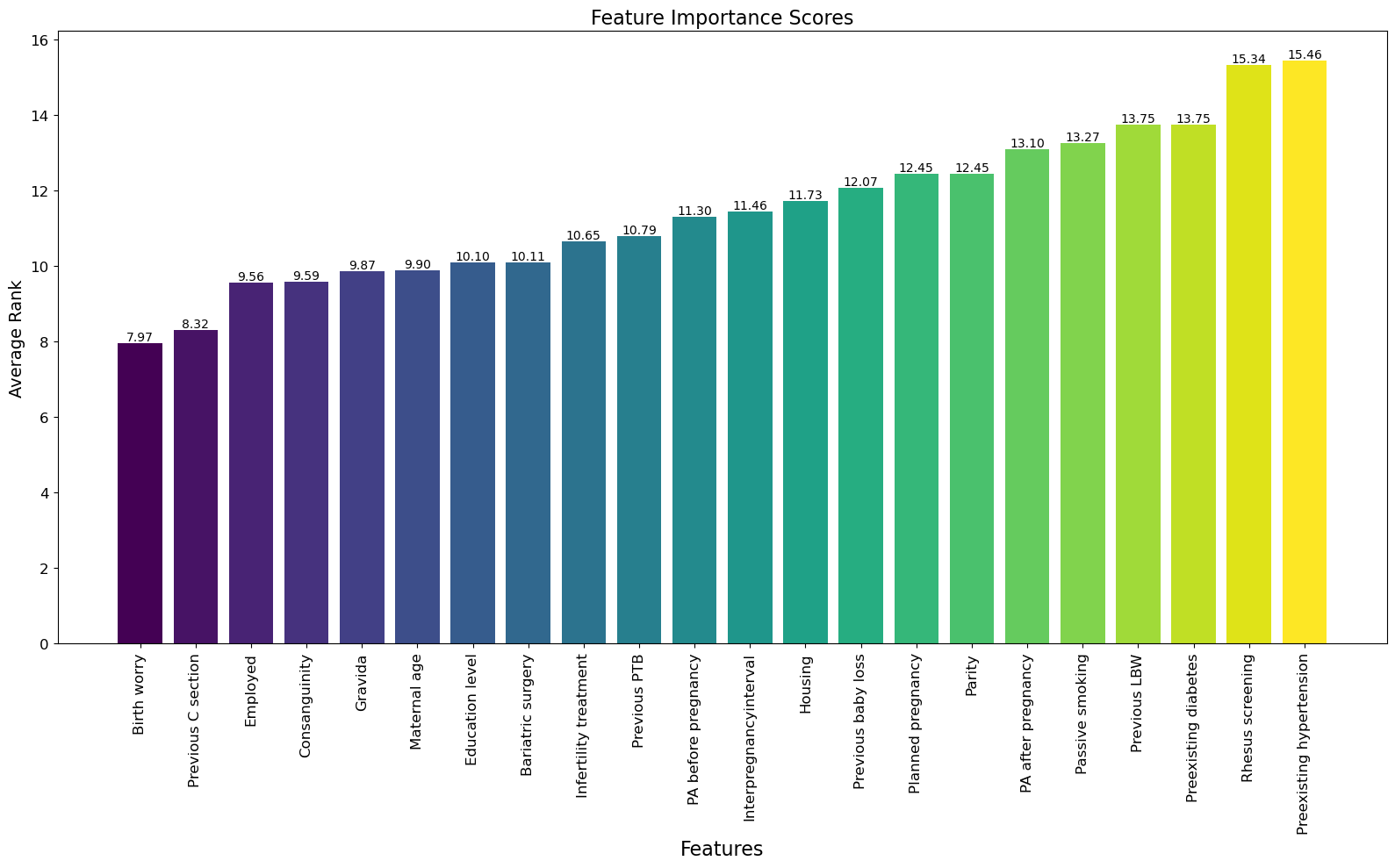


Figure 26. Average rank of all the features across all the outlier points for very low birth weight detection using Local-DIFFI

Table 26. Average rank of all the features across all the outlier points for very low birth weight detection using Local-DIFFI

| **Rank** | **Feature** | **Importance Score** |
| --- | --- | --- |
| 1 | Preexisting hypertension | 15.46 |
| 2 | Rhesus screening | 15.34 |
| 3 | Preexisting diabetes | 13.75 |
| 4 | Previous LBW | 13.75 |
| 5 | Passive smoking | 13.27 |


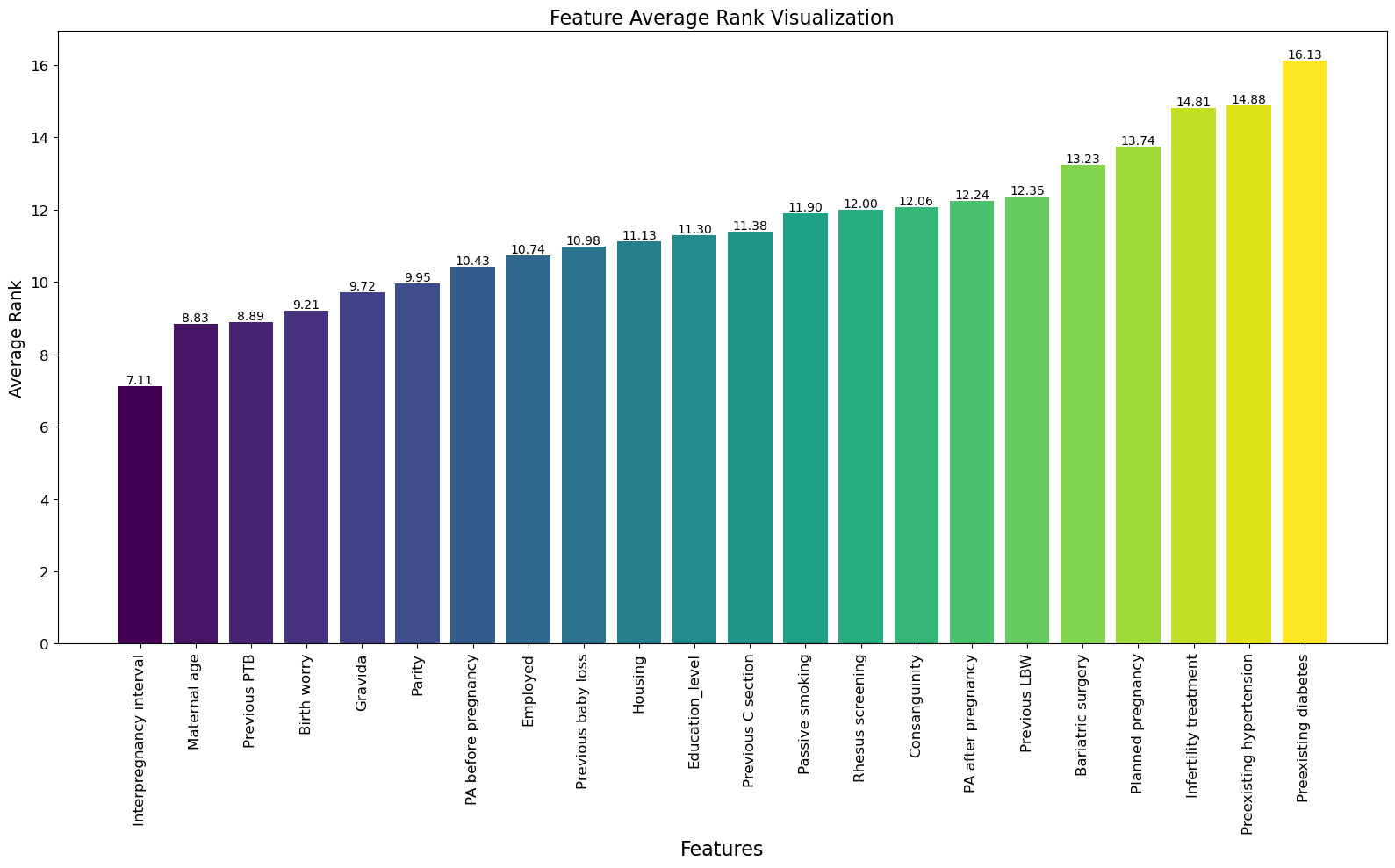


Figure 27. Average rank of all the features across all the outlier points for extreme low birth weight detection using Local-DIFFI

Table 27. Average rank of all the features across all the outlier points for extreme low birth weight detection using Local-DIFFI

| **Rank** | **Feature** | **Importance Score** |
| --- | --- | --- |
| 1 | Preexisting diabetes | 16.13 |
| 2 | Preexisting hypertension | 14.88 |
| 3 | Interfertility treatment | 14.81 |
| 4 | Planned pregnancy | 13.74 |
| 5 | Bariatric surgery | 13.23 |
